# Supplementary material for: Arterial Baroreceptors Sense Blood Pressure through Decorated Aortic Claws
Source: Cell Rep. Author manuscript; Available in PMC 2019 Dec 5. (PMC6893869; doi:10.1016/j.celrep.2019.10.040)
Supplement: 1 [file NIHMS1544238-supplement-1.pdf]

Cell Reports, Volume *29*

Supplemental Information

Arterial Baroreceptors Sense Blood Pressure through Decorated Aortic Claws

Soohong Min, Rui B. Chang, Sara L. Prescott, Brennan Beeler, Narendra R. Joshi, David E. Strochlic, and Stephen D. Liberles

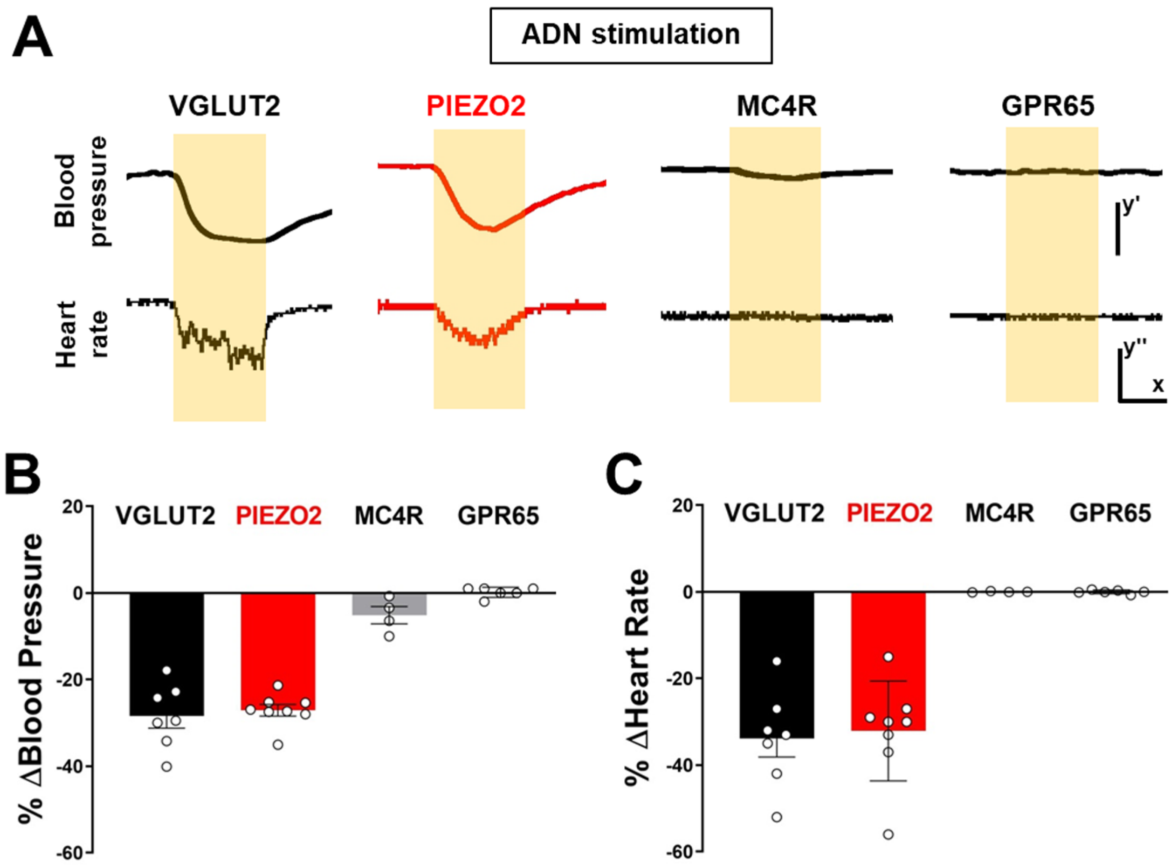

Figure S1. Optogenetic activation of sensory neurons in the aortic depressor nerve (Related to Figure 1). (A) Representative traces of blood pressure and heart rate with optogenetic stimulation (yellow shading) of the aortic depressor nerve in VGLUT2 (*Vglut2-ires-Cre; loxP-ChR2*), PIEZO2 (*Piezo2-ires-Cre; loxP-ChR2*, red), MC4R (*Mc4r-2a-Cre; loxP-ChR2*), and GPR65 (*Gpr65-ires-Cre; loxP-ChR2*) mice, scale bars: y': 20 mmHg, x: 5 sec, y'': 100 BPM. Quantifying changes in blood pressure (B) and heart rate (C) following illumination of the aortic depressor nerve in mice indicated, n: 4-8, mean ± sem.

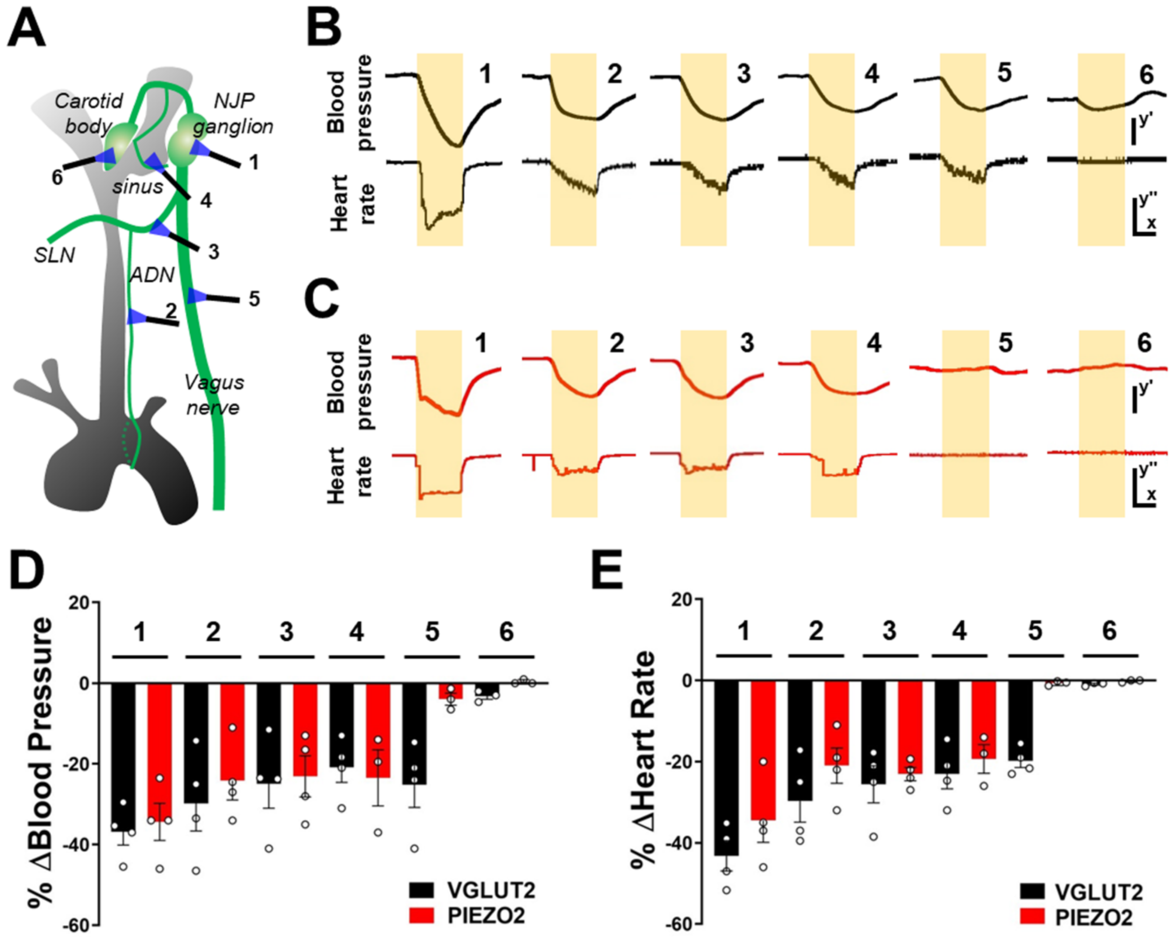

Figure S2. Nerve branch-selective optogenetics (Related to Figure 1). (A) Cartoon depicting various sites of optogenetic stimulation, including 1: NJP ganglion, 2: aortic depressor nerve (ADN), 3: superior laryngeal nerve (SLN), 4: carotid sinus, 5: vagal trunk after departure of SLN, and 6: carotid body. Representative traces of blood pressure and heart rate with optogenetic stimulation (yellow shading) occurring at sites indicated (1-6 from panel A) in *Vglut2-ires-Cre; loxP-ChR2* (B, black) and *Piezo2-ires-Cre; loxP-ChR2* (C, red) mice, scale bars: y': 20 mmHg, x: 5 sec, y'': 100 BPM. Quantifying changes in blood pressure (D) and heart rate (E) following optogenetic stimulation of *Vglut2-ires-Cre; loxP-ChR2* (black) and *Piezo2-ires-Cre; loxP-ChR2* (red) mice, n: 3-4, mean ± sem).

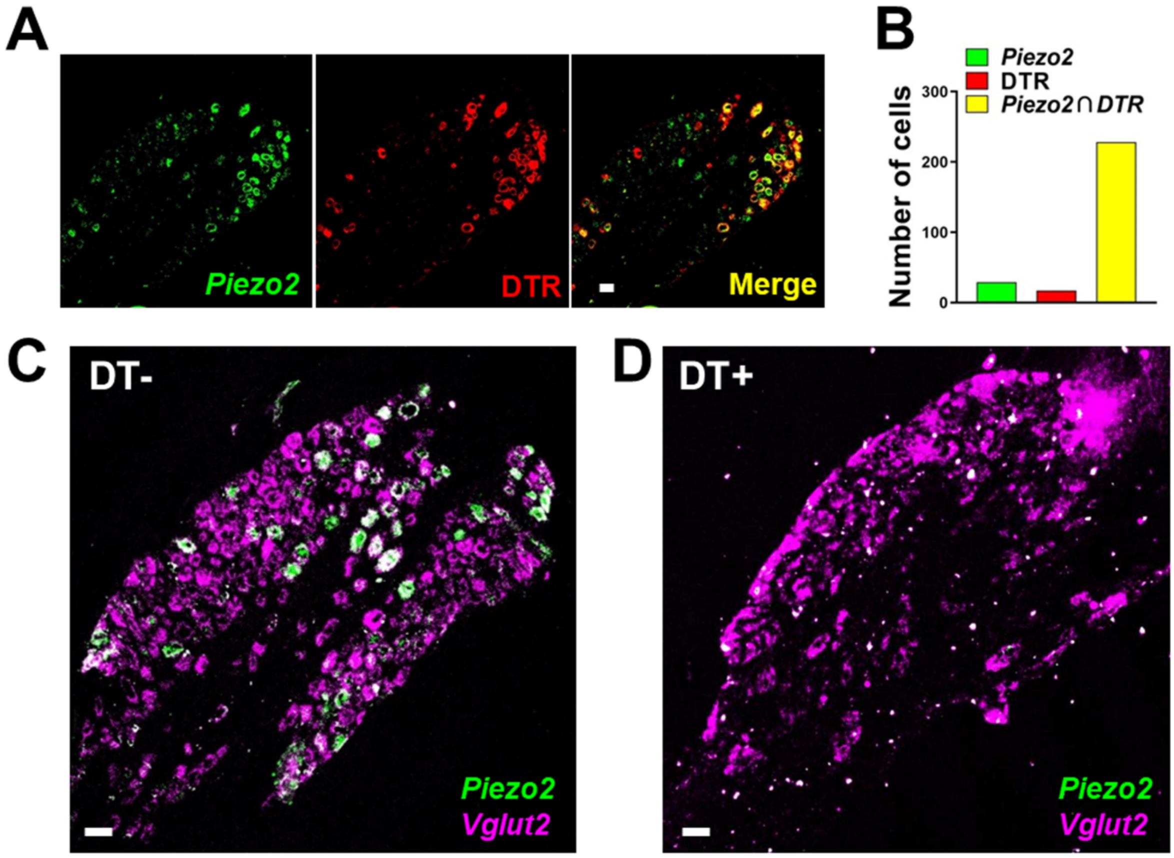

Figure S3. Assessing specificity of PIEZO2 neuron targeting in *Piezo2-ABLATE* mice (Related to Figure 2). (A) Sequential *in situ* hybridization for *Piezo2* transcript (green) and immunochemistry for DTR (red) in cryosections of vagal ganglia from *Piezo2-ires-Cre; loxP-DTR* mice, scale bar: 20 µm. (B) Counts of cells expressing only *Piezo2* (green), only DTR (red), or both (yellow), n: 5 sections from 2 mice. Two color *in situ* hybridization using cRNA probes that recognize *Piezo2* (green) and *Vglut2* (magenta) in cryosections of vagal ganglia from *Piezo2-ires-Cre; loxP-DTR* mice treated (C) without or (D) with DT, scale bar: 30 µm.

A

Blood pressure (mmHg)	Control	*Piezo2-ABLATE* (Bilateral)	*Piezo2-ABLATE* (Unilateral)
**Mean**	66.33	69.92	69.43
**Standard Error**	±4.775	±2.237	±4.657

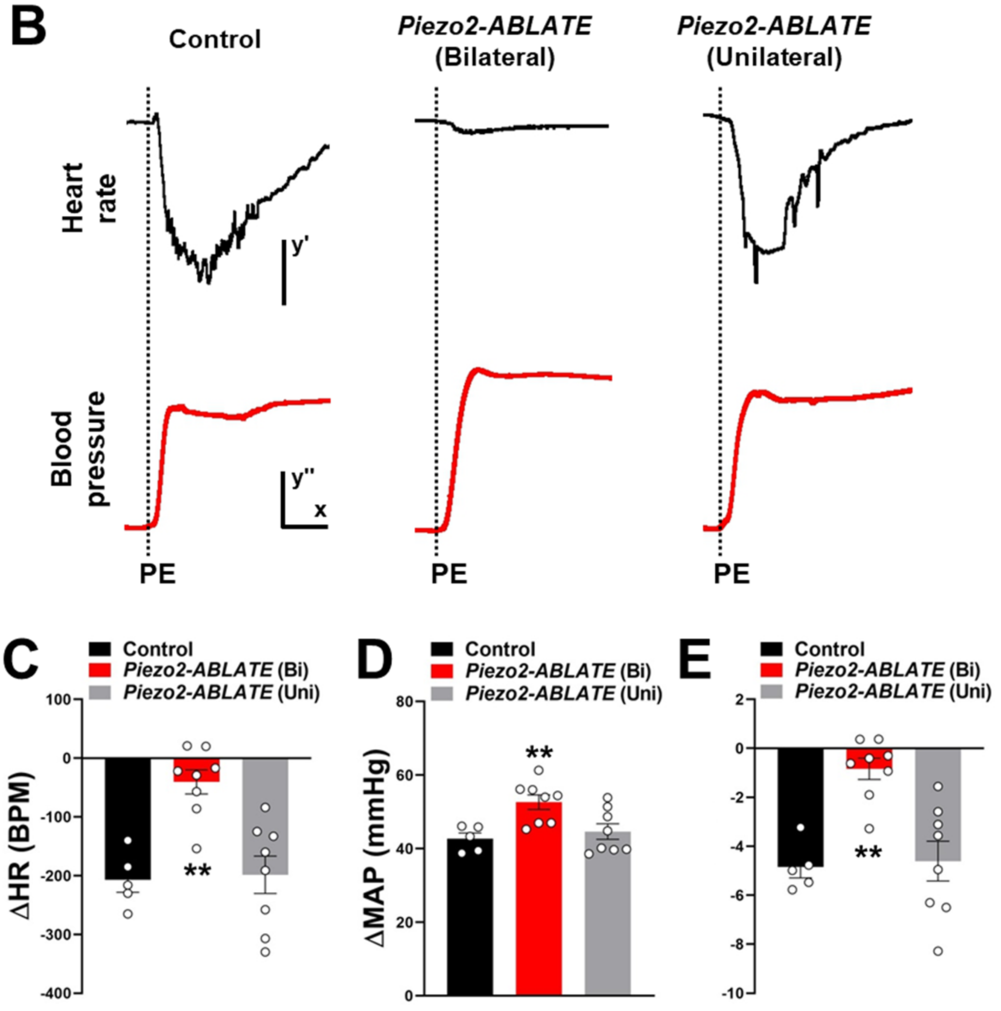

Figure S4. Loss of baroreceptor reflex requires bilateral ablation of PIEZO2 neurons in NJP ganglia (Related to Figure 2). (A) Resting blood pressure of *loxP-DTR* mice injected bilaterally with DT (control) and *Piezo2-ires-Cre; loxP-DTR* mice injected unilaterally or bilaterally with DT. (B) Assessment of baroreflex integrity in *loxP-DTR* mice injected bilaterally with DT (control), and *Piezo2-ires-Cre; loxP-DTR* mice injected unilaterally or bilaterally with DT. Representative effects of phenylephrine injection (dashed line) on blood pressure and heart rate, scale bars, y': 100 BPM, x: 10 sec, y'': 20 mmHg. Quantification of phenylephrine (PE)-induced change in heart rate or HR (C), change in mean arterial blood pressure or MAP (D), and baroreflex (E), defined as change in HR (Δ BPM) divided by change in BP (Δ mmHg), n: 5-8, mean ± sem, **p<.005.

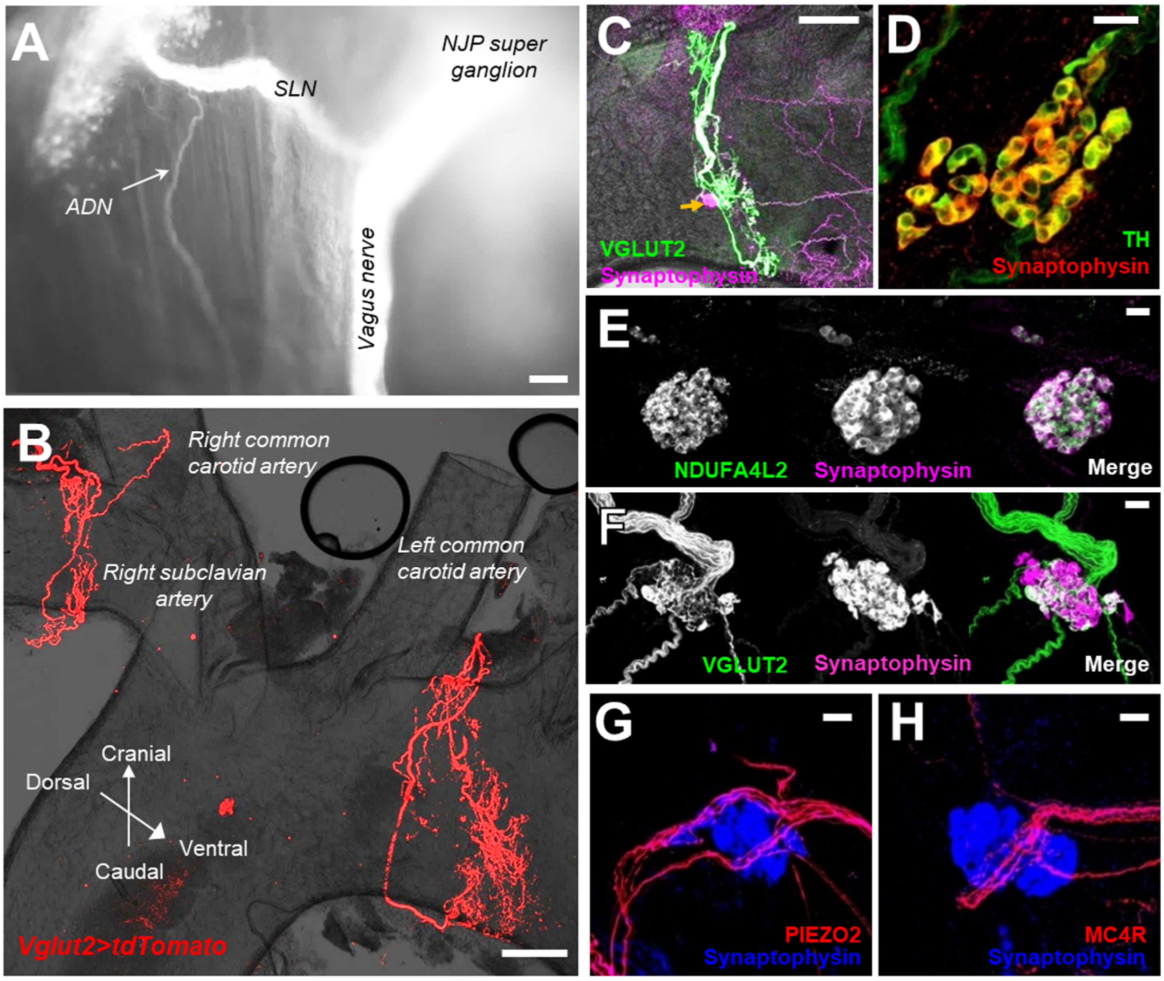

Figure S5. Visualizing innervation of the carotid sinus (Related to Figure 5). (A) Wholemount image of native fluorescence from the aortic depressor nerve (ADN) and superior laryngeal nerve (SLN) after ganglion injection of *AAV-Gfp* in wild type mice, scale bar 100 µm. (B) Wholemount anti-tdTomato immunofluorescence of the great aortic vessels in *Vglut2-ires-Cre; loxP-tdTomato* mice, scale bar 300 µm. (C) Aortic arch immunohistochemistry for tdTomato (green) and synaptophysin (magenta) after injection of *AAV-flex-tdTomato* into NJP ganglia of *Vglut2-ires-Cre* mice, yellow arrow indicates location of an aortic body, scale bar 300 µm. (D) Aortic arch immunohistochemistry for tyrosine hydroxylase (green) and synaptophysin (red), scale bar 20 µm. (E) Aortic body immunohistochemistry for NDUFA4L2 (green) and synaptophysin (magenta), scale bar 20 µm. (F) Aortic body immunohistochemistry for tdTomato (green) and synaptophysin (magenta) after injection of *AAV-flex-tdTomato* into NJP ganglia of *Vglut2-ires-Cre* mice, scale bar 20 µm. Two color immunohistochemistry for synaptophysin (blue) and tdTomato (red) to visualize neuronal fibers of passage labeled by injection of *AAV-flex-tdTomato* into NJP ganglia of *Piezo2-ires-Cre* mice (G) and *Mc4r-2a-Cre* mice (H), scale bar 20 µm.

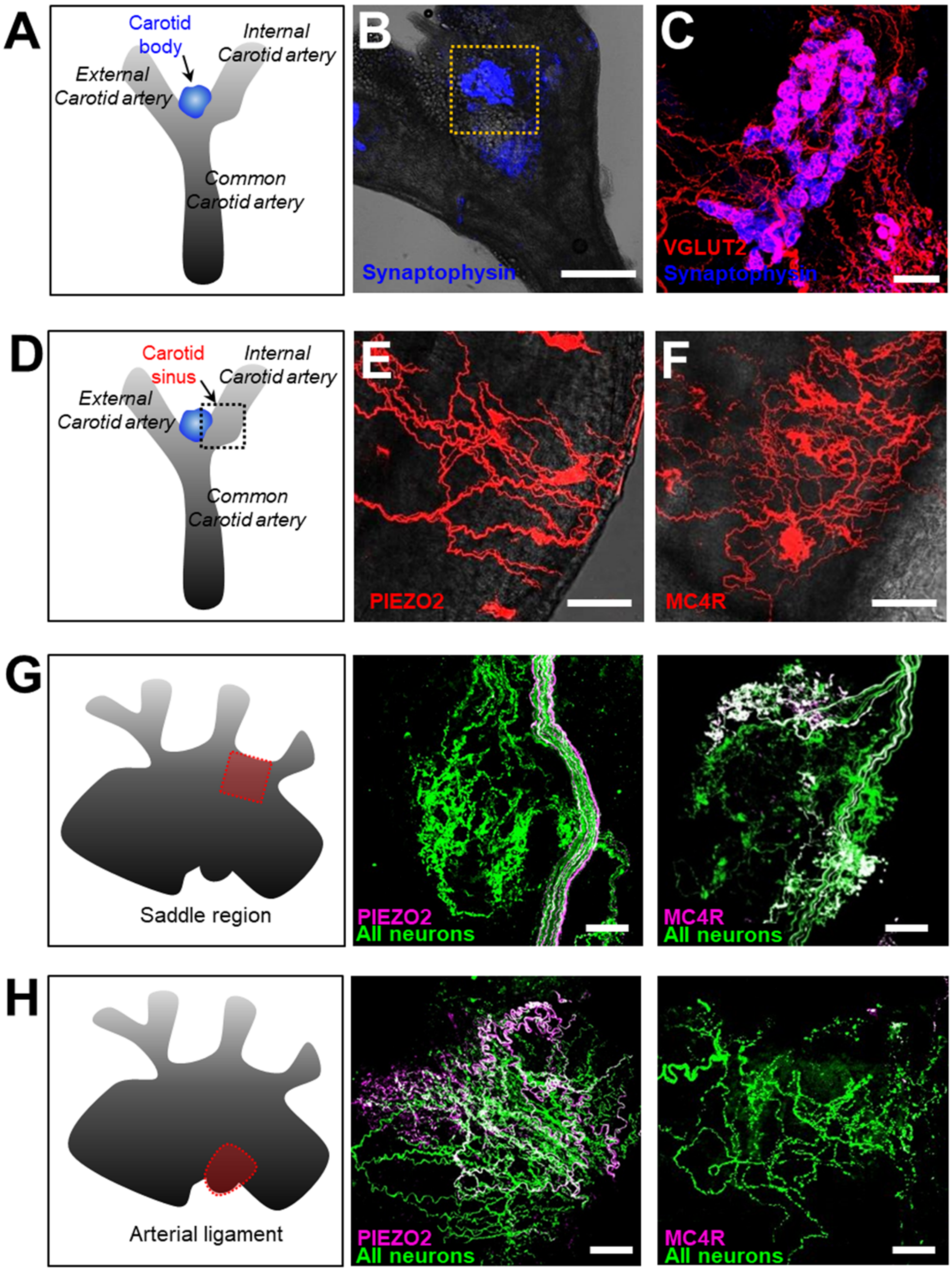

Figure S6. Neuron subtypes that innervate the carotid sinus and aortic arch (Related to Figure 5). (A, D) Cartoon depictions of the carotid sinus. Carotid glomus cells can be visualized (blue) by immunostaining for synaptophysin (B-C). Vagal afferents were visualized by immunochemistry for tdTomato (red) following injection of *AAV-flex-tdTomato* into NJP ganglia of *Vglut2-ires-Cre* mice (C)*, Piezo2-ires-Cre* mice (E), and *Mc4r-2a-Cre* mice (F), scale bars: 400 μm for B; 20 μm for C; 100 μm for E-F. Boxed region in B depicts regions of analysis for panels C, while boxed region in D depicts region of analysis for E-F. Representative images used for quantitative analysis of flower spray terminals in the aortic saddle region (G) and end-net endings in the arterial ligament (H). Vagal afferents were visualized by immunochemistry following injection of Cre-independent *AAV-Gfp* (green, ALL NEURONS) and *AAV-flex-tdTomato* (magenta) into NJP ganglia of *Piezo2-ires-Cre* (PIEZO2) or *Mc4r-2a-Cre* (MC4R) mice, scale bar 30 μm.

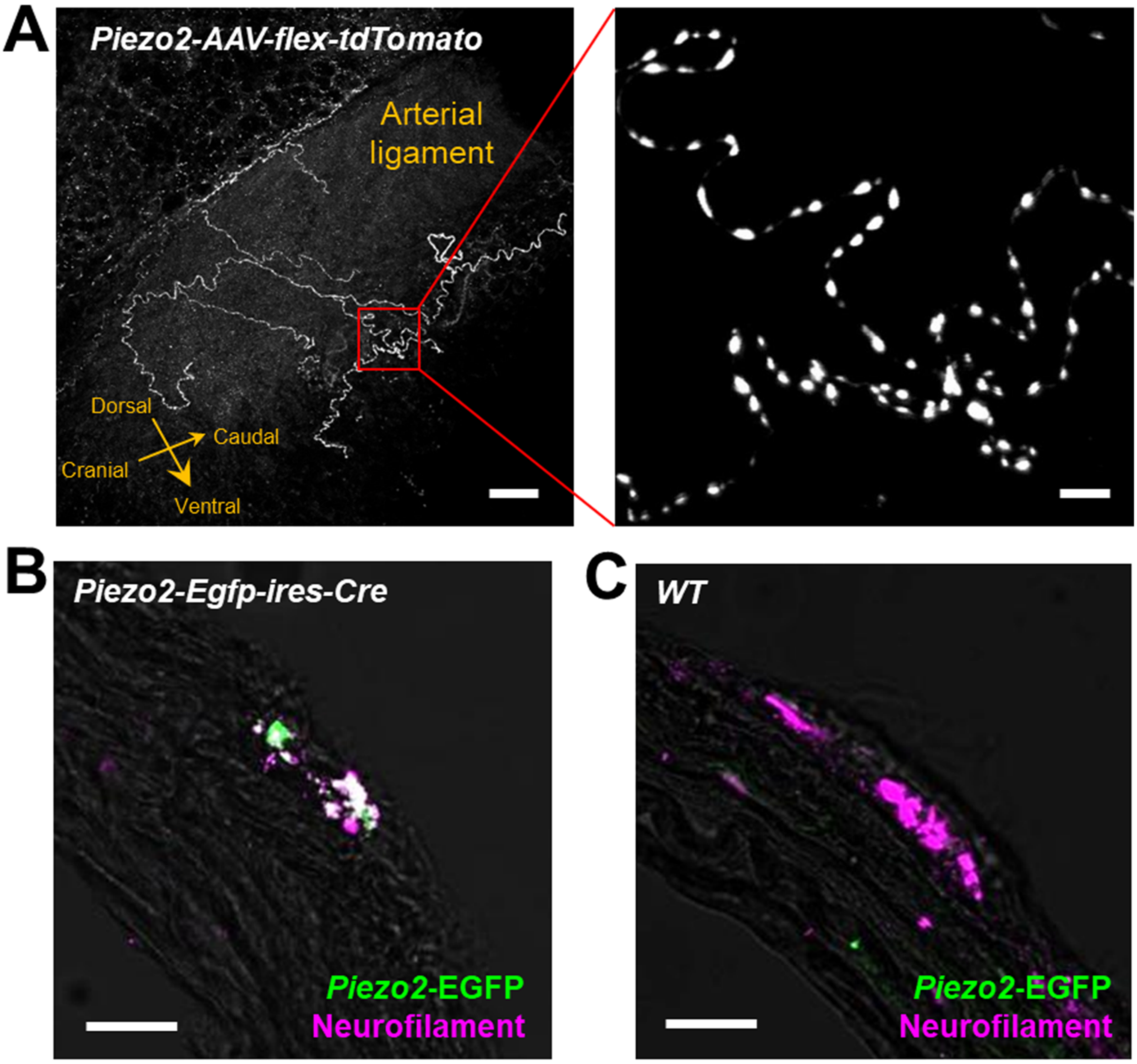

Figure S7. Sensory neuron innervation of the arterial ligament (Related to Figure 7). (A) Vagal afferents were visualized in the arterial ligament by immunochemistry for tdTomato following injection of *AAV-flex-tdTomato* into NJP ganglia of *Piezo2-ires-Cre* mice, scale bars, left: 30 µm, right: 10 µm. The right panel is a high magnification, partial Z-stack image from a region (red box) of the left image. Immunohistochemistry for GFP (green) and neurofilament (magenta) in arterial ligament of (B) *Piezo2-ires Cre* and (C) wild type mice. *Piezo2-ires-Cre* mice express a PIEZO2-GFP fusion protein from the endogenous *Piezo2* locus, scale bars: 10 µm.
