# Supplementary material for: Arterial Baroreceptors Sense Blood Pressure through Decorated Aortic Claws
Source: Cell Rep. Author manuscript; Available in PMC 2019 Dec 5. (PMC6893869; doi:10.1016/j.celrep.2019.10.040)
Supplement: 2 [file NIHMS1544238-supplement-2.pdf]

Cell Reports

Article

Arterial Baroreceptors Sense Blood Pressure through Decorated Aortic Claws

Graphical Abstract

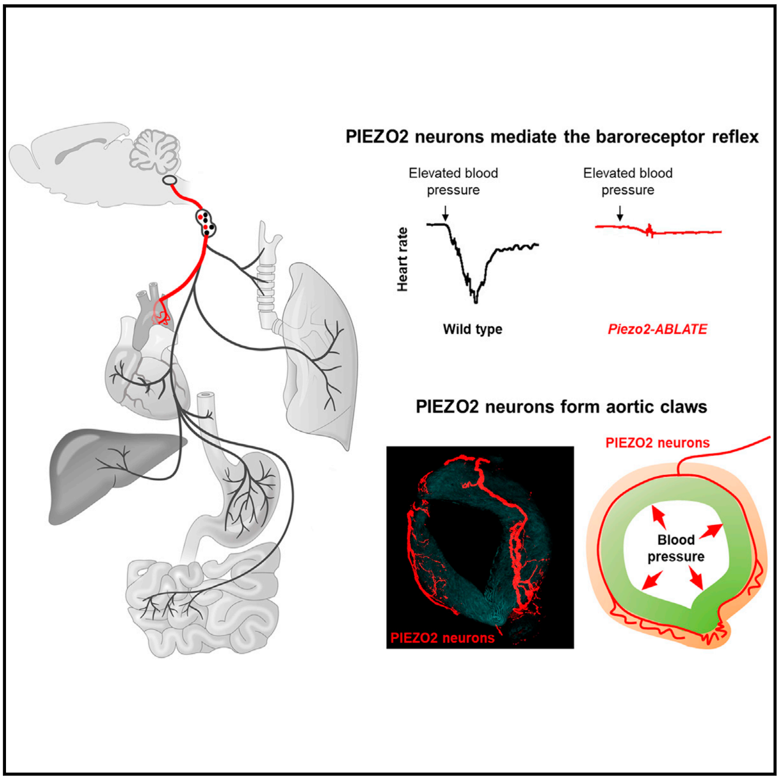

Authors

Soohong Min, Rui B. Chang, Sara L. Prescott, Brennan Beeler, Narendra R. Joshi, David E. Strochlic, Stephen D. Liberles

Correspondence

stephen_liberles@hms.harvard.edu

In Brief

Min et al. use genetic approaches to reveal how neurons sense blood pressure. Elevated blood pressure evokes a classic neuronal reflex (the baroreceptor reflex), found here to require PIEZO2 neurons. To sense blood pressure, PIEZO2 neurons form large claws that surround the aorta and are decorated with mechanosensory endings.

Highlights

- Genetic mapping, ablation, and control of artery-innervating PIEZO2 neurons
- PIEZO2 neurons of vagal and petrosal ganglia mediate the baroreceptor reflex
- Baroreceptor neurons form macroscopic claws that surround the aortic arch
- PIEZO2 neurons sense blood pressure with end-net endings

Min et al., 2019, Cell Reports *29*, 2192–2201
November 19, 2019 © 2019 The Authors.
https://doi.org/10.1016/j.celrep.2019.10.040

CellPress

OPEN ACCESS
CellPress

Arterial Baroreceptors Sense Blood Pressure through Decorated Aortic Claws

Soohong Min,[1] Rui B. Chang,[1] Sara L. Prescott,[1] Brennan Beeler,[1] Narendra R. Joshi,[1] David E. Strochlic,[1] and Stephen D. Liberles[1,2,*]
[1]Howard Hughes Medical Institute, Department of Cell Biology, Harvard Medical School, Boston, MA 02115, USA
[2]Lead Contact
*Correspondence: stephen_liberles@hms.harvard.edu
https://doi.org/10.1016/j.celrep.2019.10.040

SUMMARY

Mechanosensory neurons across physiological systems sense force using diverse terminal morphologies. Arterial baroreceptors are sensory neurons that monitor blood pressure for real-time stabilization of cardiovascular output. Various aortic sensory terminals have been described, but those that sense blood pressure are unclear because of a lack of selective genetic tools. Here, we find that all baroreceptor neurons are marked in *Piezo2-ires-Cre* mice and then use genetic approaches to visualize the architecture of mechanosensory endings. Cre-guided ablation of vagal and glossopharyngeal PIEZO2 neurons eliminates the baroreceptor reflex and aortic depressor nerve effects on blood pressure and heart rate. Genetic mapping reveals that PIEZO2 neurons form a distinctive mechanosensory structure: macroscopic claws that surround the aortic arch and exude fine end-net endings. Other arterial sensory neurons that form flower-spray terminals are dispensable for baroreception. Together, these findings provide structural insights into how blood pressure is sensed in the aortic vessel wall.

INTRODUCTION

Sensory neurons densely innervate the great vessels of the vascular system, providing essential moment-by-moment feedback for control of heart rate, blood pressure, and respiration. One classic cardiovascular reflex is the baroreceptor reflex, where elevated blood pressure instantaneously triggers compensatory decreases in cardiovascular output to steady blood flow to the brain and body (Benarroch, 2008; Brown, 1980; Kirchheim, 1976; Kumada et al., 1990; Wehrwein and Joyner, 2013). However, a description of baroreceptor morphology is lacking and is needed to understand mechanisms of force sensation by neurons within the arterial wall.

Blood pressure sensation occurs at several hotspots within the vascular system. Afferents of the vagus nerve (cranial nerve 10) and glossopharyngeal nerve (cranial nerve 9) target the aortic arch and carotid sinus, respectively. In mouse, vagal and glossopharyngeal ganglia are fused into nodose/jugular/petrosal (NJP) superganglia. Vagal sensory neurons access the aorta through a fine nerve branch termed the aortic depressor nerve, while glossopharyngeal neurons access the carotid sinus through the carotid sinus nerve. Afferents from the left nodose ganglion innervate the apex of the aortic arch between the left common carotid and left subclavian arteries, while afferents from the right nodose ganglion innervate slightly higher in the thorax, on the right subclavian artery near its departure point from the innominate artery. The aortic depressor and carotid sinus nerves consist of co-fasciculating fibers, including both mechanosensory and chemosensory afferents.

The baroreceptors innervate specialized areas of blood vessel wall that are unusually elastic, because of local thinning of smooth muscle as well as altered abundance of elastin and collagen fibers (Kirchheim, 1976; Rees, 1968). Blood pressure pulses that occur with each heartbeat radially stretch the elastic vessel wall, and this arterial distension in turn activates mechanosensitive neurons (Kirchheim, 1976; Kumada et al., 1990). Neuronal inputs inform about stretch magnitude, pulse frequency, and mean arterial pressure (MAP) and can be bidirectionally modulated, allowing appropriate reflex action to both decreases and increases in blood pressure (Kirchheim, 1976; Kumada et al., 1990). Baroreceptor neurons are long aorta-to-brain sensory neurons that transmit inputs directly to the brainstem. In response to baroreceptor activation, parallel neural pathways are engaged that decrease sympathetic output and enhance parasympathetic output, ultimately lowering heart rate and blood pressure (Andresen and Kunze, 1994; Spyer, 1989).

PIEZO proteins function as mechanosensitive ion channels critical for neuronal sensation of blood pressure and the baroreceptor reflex (Zeng et al., 2018). PIEZOs are enormous ion channels that are intrinsically gated by force in the absence of auxiliary factors and are essential for normal touch sensation, proprioception, and airway stretch sensation (Nonomura et al., 2017; Ranade et al., 2014; Woo et al., 2015). PIEZO2 is expressed in a subset of sensory neurons in vagal (nodose/jugular) and glossopharyngeal (petrosal) ganglia (Chang et al., 2015). Optogenetic activation of vagal afferents containing PIEZO2 interrupts breathing (Chang et al., 2015; Nonomura et al., 2017), as PIEZO2 mediates airway stretch sensation underlying the Hering-Breuer inspiratory reflex (Nonomura et al., 2017), and also decreases heart rate and blood pressure (Zeng et al., 2018), signatures of the baroreceptor reflex. Knockout mice lacking *Piezo1* and *Piezo2* in *Phox2b*-expressing cells, which include sensory neurons of the nodose and inferior petrosal ganglia as well as other

This is an open access article under the CC BY license (http://creativecommons.org/licenses/by/4.0/).

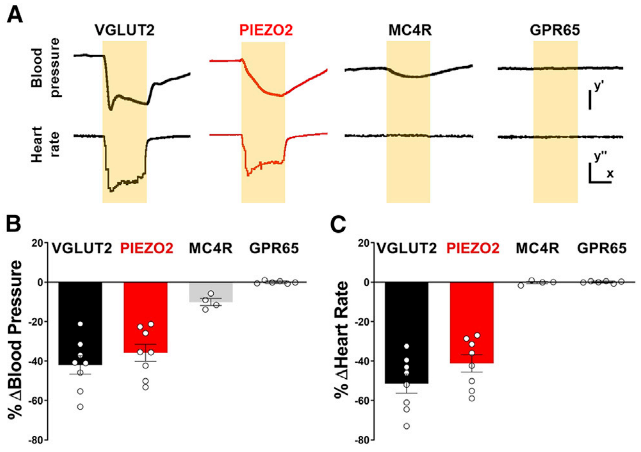

Figure 1. Optogenetic Control of Heart Rate and Blood Pressure

(A) Representative traces of mean arterial blood pressure (BP) and heart rate (HR) following focal illumination (yellow shading) of the vagus nerve in anesthetized VGLUT2 (*Vglut2-ires-Cre; loxP-ChR2*), PIEZO2 (*Piezo2-ires-Cre; loxP-ChR2*, red), MC4R (*Mc4r-2a-Cre; loxP-ChR2*), and GPR65 (*Gpr65-ires-Cre; loxP-ChR2*) mice. Scale bars: y′, 20 mmHg; x, 5 s; y″, 100 BPM.

(B and C) Light-induced changes in blood pressure (B) and heart rate (C) were quantified over the 10 s trial and compared with the immediate 10 s pre-stimulus period (n = 4–8; mean ± SEM).

See also Figures S1 and S2.

cell types, fail to display reflexive heart rate control or aortic depressor nerve responses after induced vasoconstriction and also display labile hypertension and increased blood pressure variability (Zeng et al., 2018). Together, these studies indicated that PIEZOs are essential for baroreceptor function.

Knockout of both *Piezo1* and *Piezo2*, but neither one alone, eliminates the baroreceptor reflex (Zeng et al., 2018). In contrast, vagal responses to airway stretch and certain dorsal root ganglia responses require only PIEZO2 (Nonomura et al., 2017; Ranade et al., 2014; Woo et al., 2015). A few models are possible to explain the sufficiency of either PIEZO1 or PIEZO2 for blood pressure sensation. It is possible that PIEZO1 and PIEZO2 mark discrete types of baroreceptor afferents that display subtle differences in response properties, such as response threshold, response kinetics, or adaptation rate. Baroreceptor neurons with different response properties and conduction velocities have been reported in electrophysiological studies (Fidone and Sato, 1969; Kumada et al., 1990). Alternatively, or in addition, PIEZO1 and PIEZO2 may collaborate in the same class of sensory neuron to sense arterial stretch.

Understanding the anatomical arrangement of arterial mechanoreceptors is essential for appreciating how these neurons transduce force. However, the morphology of force-sensing terminals marked by PIEZO expression also remains unresolved. The structures of neuronal endings in the carotid sinus and aortic arch have been extensively studied using both light microscopy and electron microscopy (Aumonier, 1972; Krauhs, 1979). However, early studies into baroreceptor structure assumed that the rat aortic depressor nerve consisted exclusively of baroreceptors, so resulting analyses lacked cellular specificity. More recent work, in rat and other species, indicated that aortic sensory neurons displayed three principal terminal morphologies: flower-spray endings, end-net terminals, and glomus cell contacts (Cheng et al., 1997). Afferents near glomus cells have been presumed to function as chemoreceptors, but it has been unclear whether flower-spray endings and end-net endings are different types of baroreceptors or whether they serve alternative sensory functions. Approaches to selectively activate or eliminate each terminal type have been needed to distinguish their physiological roles.

Here, optogenetics and cell ablation approaches demonstrated that baroreceptor neurons are comprehensively marked in *Piezo2-ires-Cre* mice. Genetic mapping of aortic terminals in *Piezo2-ires-Cre* mice then revealed the peripheral morphology of arterial baroreceptors. We find that blood pressure is sensed by mechanosensory neurons with macroscopic claws that circumnavigate the aortic arch and are laterally adorned with end-net endings.

RESULTS

Genetic Identification of Baroreceptor Neurons through Optogenetics

In prior studies, we generated a large collection of Cre knockin mice that target different subtypes of vagal sensory neurons and adapted genetic approaches for cell-specific neural mapping and optogenetics (Chang et al., 2015; Williams et al., 2016). We described vagal sensory neuron types that innervate the airways and powerfully control breathing (Chang et al., 2015) and others that monitor and control the digestive system (Williams et al., 2016). Here, we used optogenetic approaches to identify neurons that affected cardiovascular physiology.

We drove channelrhodopsin expression in peripheral sensory neurons using Cre knockin mice and a Cre-dependent channelrhodopsin allele (*loxP-ChR2*). We then activated sensory neurons by illumination of NJP ganglia or particular nerve branches. All vagal and glossopharyngeal sensory neurons are thought to release glutamate, and in *Vglut2-ires-Cre* mice, >99% of NJP sensory neurons express Cre-dependent reporter genes from the *Rosa26* locus (Chang et al., 2015). Acute optogenetic activation of all vagal sensory neurons in *Vglut2-ires-Cre; loxP-ChR2* mice affected several major physiological systems (Chang et al., 2015; Williams et al., 2016) and caused decreases in heart rate (−52.0%) and blood pressure (−42.0%) (Figure 1).

We next performed similar experiments to activate small subsets of NJP sensory neurons. We identified one neuron type, PIEZO2 neurons marked in *Piezo2-ires-Cre* mice, which evoked physiological changes in heart rate and blood pressure comparable with the baroreceptor reflex. Little or no effect on cardiovascular output was observed following stimulation of vagal MC4R neurons, which also innervate the aorta (see below), and vagal GPR65 neurons, which do not. As reported previously (Zeng et al., 2018), focal illumination of NJP soma in *Piezo2-ires-Cre; loxP-ChR2* mice powerfully decreased both heart rate (−41.2%) and blood pressure (−35.8%). Similar experiments in *Mc4r-2a-Cre; loxP-ChR2* mice or *Gpr65-ires-Cre; loxP-ChR2* mice had little or no impact on heart rate (MC4R, −0.0%; GPR65, +0.1%) or blood pressure (MC4R, −10.0%; GPR65, −0%). Furthermore, decreases in heart rate (−32.1%) and blood pressure (−27.1%) were observed in optogenetic experiments involving illumination of only the aortic

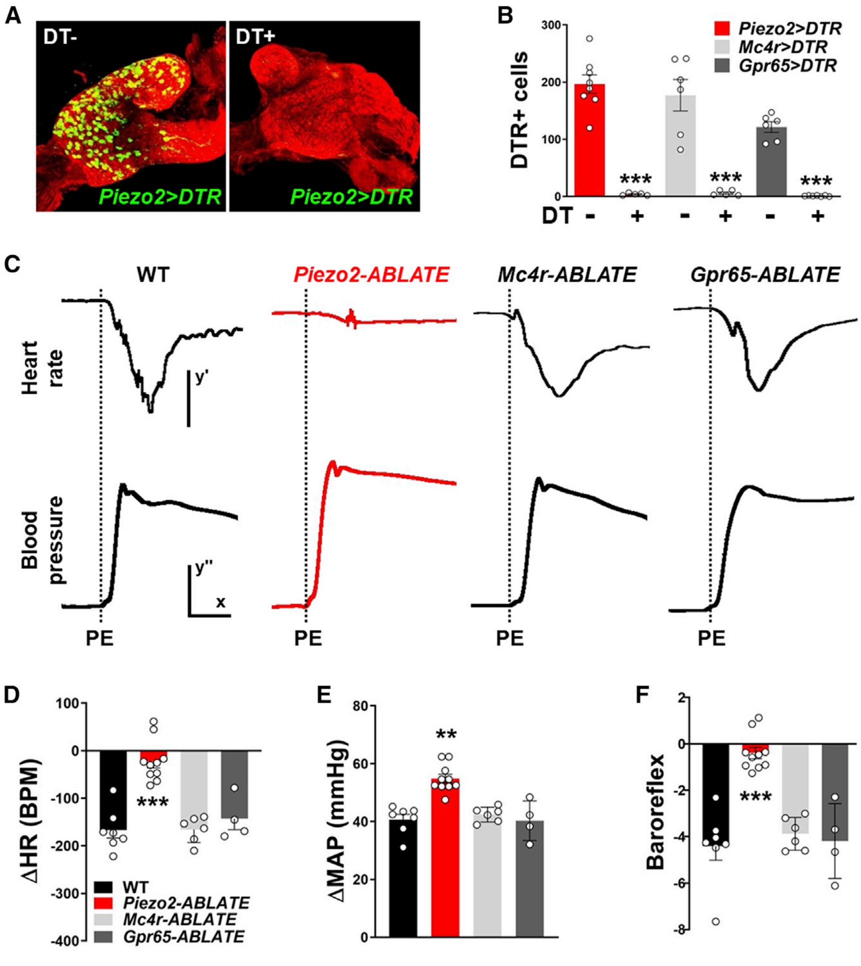

Figure 2. PIEZO2 Neurons Mediate the Baroreceptor Reflex

(A) Assessing efficiency of DT-guided neuron ablation. Immunochemistry for DTR (green) and neurofilament (red) in NJP ganglia of *Piezo2-ires-Cre; loxP-DTR* mice with (right) or without (left) injection of DT.

(B) The number of DTR-expressing cells in NJP ganglia of *Piezo2-ires-Cre; loxP-DTR* (red), *Mc4r-2a-Cre; loxP-DTR* (gray), and *Gpr65-ires-Cre; loxP-DTR* (black) mice with (+) or without (−) DT injection. mean ± SEM. ***$p < 0.0005$, unpaired t test.

(C) Assessment of baroreflex integrity in wild-type, *Piezo2-ABLATE*, *Mc4r-ABLATE*, and *Gpr65-ABLATE* mice. Representative effects of phenylephrine (PE) injection (dashed line) on MAP and heart rate. Scale bars: y′, 100 BPM; x, 10 s; y″, 20 mmHg.

(D–F) Quantification of phenylephrine (PE)-induced change in heart rate (HR) (D), change in MAP (E), and baroreflex (F), defined as change in HR (ΔBPM) divided by change in BP (ΔmmHg); n = 4–10; mean ± SEM.

**$p < 0.005$ and ***$p < 0.0005$, ANOVA (Dunnett's multiple-comparison test).

See also Figures S3 and S4.

depressor nerve in *Piezo2-ires-Cre; loxP-ChR2* mice (Figures S1 and S2). These studies reveal that PIEZO2 neurons of NJP ganglia, but not other neuron types analyzed, evoke baroreceptor-associated physiological changes.

Selective Ablation of PIEZO2 Neurons Eliminates the Baroreceptor Reflex

Of the neuronal subtypes examined, only PIEZO2 neurons evoked both a decrease in heart rate and blood pressure reminiscent of the baroreceptor reflex. It is possible that there are multiple types of baroreceptors (for example, some might express only PIEZO1) and that we simply lack genetic tools to visualize other relevant mechanosensory neurons of the aortic depressor nerve. Alternatively, it is possible that most or all baroreceptor terminals are marked in *Piezo2-ires-Cre* mice. To investigate these possibilities, we asked whether ablation of PIEZO2 neurons in NJP ganglia affected the baroreceptor reflex.

We used a genetic strategy involving targeted expression of diphtheria toxin (DT) to ablate Cre-expressing NJP sensory neurons. Mouse cells are normally resistant to DT-induced apoptosis but can be rendered susceptible by expression of the DT receptor (DTR) (Saito et al., 2001). A Cre-dependent DTR allele (*loxP-DTR*) has been widely used for conditional ablation of various Cre-expressing cells in the body and brain (Buch et al., 2005), including in the vagus nerve (Tränkner et al., 2014). We generated *Piezo2-ires-Cre; loxP-DTR*, *Mc4r-2a-Cre; loxP-DTR*, and *Gpr65-ires-Cre; loxP-DTR* mice and verified that DTR expression was largely driven to the correct neurons (Figures S3A and S3B). Bilateral DT injections were performed directly into NJP ganglia (Figures 2A and 2B), resulting in targeted killing of 98% of DTR-expressing neurons in NJP ganglia of *Piezo2-ires-Cre; loxP-DTR* mice (*Piezo2-ABLATE* mice), 96.7% in *Mc4r-2a-Cre; loxP-DTR* mice (*Mc4r-ABLATE* mice), and 99.1% in *Gpr65-ires-Cre; loxP-DTR* mice (*Gpr65-ABLATE* mice). We also observed a loss of *Piezo2* transcript in NJP superganglia of *Piezo2-ABLATE* mice (Figures S3C and S3D). We note that similar ablations were not possible in *Vglut2-ires-Cre; loxP-DTR* mice that died after bilateral DT injection, apparently because of respiratory distress. DT-induced cell death was highly efficient, and *Piezo2-ABLATE*, *Mc4r-ABLATE*, and *Gpr65-ABLATE* mice survived the procedure.

Next, we assessed the integrity of the baroreceptor reflex after ablating different peripheral sensory neurons (Figures 2C–2E). A commonly used method for evoking the baroreceptor reflex involves intravenous injection of phenylephrine to induce vasoconstriction (Wehrwein and Joyner, 2013). Phenylephrine injection elevated MAP (+40.9 mmHg) in wild-type mice, and elevated blood pressure, in turn, caused a compensatory decrease in heart rate (−164 beats per minute [BPM]) through the baroreceptor reflex. Phenylephrine injection in *Mc4r-ABLATE* mice and *Gpr65-ABLATE* mice caused similar increases in MAP (+43.7 and +40.5 mmHg) and similar subsequent drops in heart

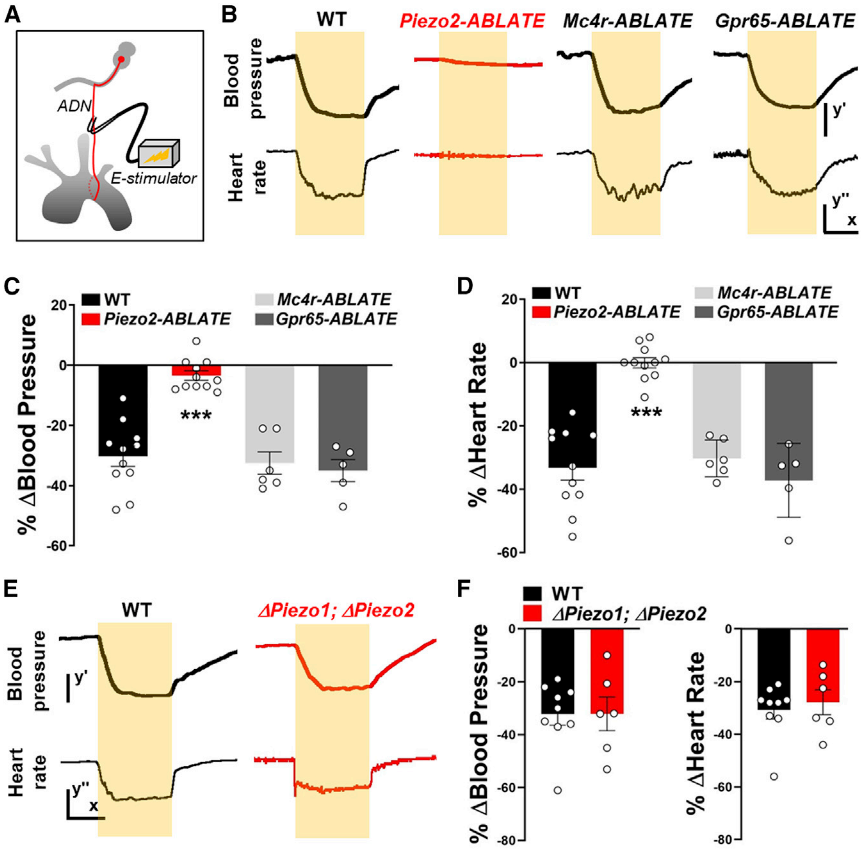

Figure 3. Loss of Aortic Depressor Nerve Function after PIEZO2 Neuron Ablation

(A) Cartoon depicting electrical stimulation of the aortic depressor nerve.

(B) Representative traces of MAP and heart rate (scale bars: y′, 20 mmHg; x, 10 s; y″, 50 BPM) before, during (yellow bars), and after electrical stimulation of the aortic depressor nerve in wild-type (WT), *Piezo2-ABLATE*, *Mc4r-ABLATE*, and *Gpr65-ABLATE* mice.

(C and D) Quantifying changes in (C) blood pressure and (D) heart rate after aortic depressor nerve stimulation in mouse lines indicated (n = 5–11; mean ± SEM; ***p < 0.0005, ANOVA [Dunnett's multiple-comparison test]).

(E and F) Representative traces (E) and quantification (F) showing that stimulation of the aortic depressor nerve yielded similar changes in MAP and heart rate (scale bars: y′, 20 mmHg; x, 10 s; y″, 50 BPM) in wild-type (WT) and *Phox2b-Cre; loxP-Piezo1; loxP-Piezo2* (Δ*Piezo1*; Δ*Piezo2*) mice (n = 6–9; mean ± SEM).

rate (−160 and −138 BPM), indicating that these neuron populations were dispensable for the baroreceptor reflex. In contrast, phenylephrine injection in *Piezo2-ABLATE* mice caused an exaggerated increase in blood pressure (+54.9 mmHg) and, despite this increase, caused a severely muted bradycardia response (−22 BPM). The small residual effect on heart rate could be due to incomplete ablation of PIEZO2 neurons by DT or a minor contribution from another afferent type. Both the enhanced blood pressure responses and impaired heart rate responses to phenylephrine injection are consistent with a striking loss of baroreceptor reflex function (Figure 2F). Unilateral DT injection in *Piezo2-ires-Cre; loxP-DTR* mice did not impair the baroreceptor reflex (Figure S4); the requirement for bilateral neuron ablation suggests that observed effects were not due to leakage of DT from the injection site and subsequent ablation of remote Cre-expressing cells. Together, these data show that in the absence of PIEZO2 NJP sensory neurons, other cell types are not sufficient to mediate a normal baroreflex. The dramatic reduction of the baroreflex in *Piezo2-ABLATE* mice indicates that NJP neurons marked in *Piezo2-ires-Cre* mice are the principal blood pressure sensors in the arterial wall.

Loss of Cardiovascular Control by the Aortic Depressor Nerve in the Absence of PIEZO2 Neurons

Next, we asked whether the aortic depressor nerve might contain sensory neurons other than PIEZO2-expressing baroreceptors that are relevant for heart rate and blood pressure control. In wild-type mice, electrical stimulation of the aortic depressor nerve caused significant decreases in blood pressure (−30.2%) and heart rate (−31.2%). Next, we asked whether electrical stimulation-evoked responses were lost after targeted neuron ablation; these experiments involved unilateral DT injections and ipsilateral nerve stimulation (Figures 3A–3D). Ablation of MC4R or GPR65 neurons had no effect on cardiovascular parameters analyzed, as electrical stimulation of the aortic depressor nerve in *Mc4r-ABLATE* mice and *Gpr65-ABLATE* mice caused comparable decreases in blood pressure (−32.5% and −35.7%) and heart rate (−35.7% and −35.8). In contrast, aortic depressor nerve stimulation in *Piezo2-ABLATE* mice caused little or no change in blood pressure (−4.2%) or heart rate (−0.4%). Thus, aortic terminals that persist after removal of PIEZO2 afferents are unable to alter blood pressure and heart rate at levels comparable with the baroreceptor reflex. We also note that electrical stimulation of the aortic depressor nerve in *Phox2b-Cre; loxP-Piezo1; loxP-Piezo2* mice, which lack a normal baroreceptor reflex (Zeng et al., 2018), still dampened heart rate and blood pressure to levels observed in wild-type mice (Figures 3E and 3F). Thus, neural circuitry underlying the baroreceptor reflex, from brainstem afferent terminals to motor output, is intact in knockout mice lacking PIEZO1 and PIEZO2 in *Phox2b*-expressing cells. Taken together, optogenetic approaches and cell ablation data indicate that PIEZO2 neurons of NJP ganglia are the principal mediators of the baroreceptor reflex.

Visualizing Baroreceptor Terminals in the Aortic Arch

We previously developed a genetic approach based on Cre/loxP technology to map the projections of specified vagal sensory neurons (Chang et al., 2015; Williams et al., 2016). Briefly, Cre-dependent adeno-associated viruses (AAVs) encoding fluorescent reporters weredirectly injected into vagal ganglia of Cre knockin

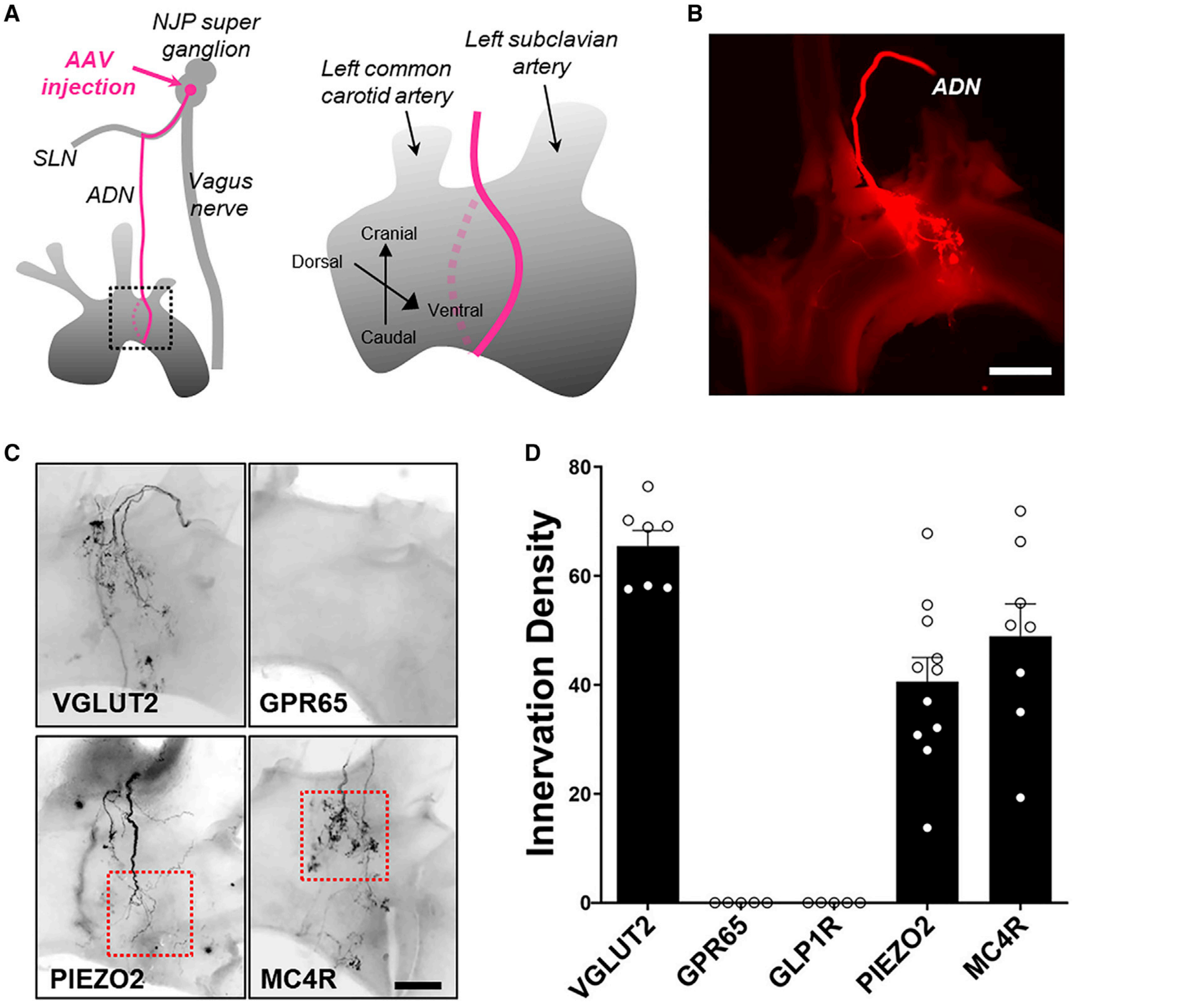

Figure 4. Visualizing Arterial Terminals by AAV Mapping

(A) Cartoon depicting AAV injection technique (left) and region of analysis (right) of aortic arch innervation by the aortic depressor nerve (ADN).

(B) Whole-mount image (native fluorescence) of aortic arch following *AAV-flex-tdTomato* injection into NJP ganglia of *Vglut2-ires-Cre* mice; scale bar, 500 μm.

(C) Whole-mount image of aortic arch immunofluorescence following *AAV-flex-tdTomato* injection into NJP ganglia of *Vglut2-ires-Cre*, *Piezo2-ires-Cre*, *Mc4r-ires-Cre*, and *Gpr65-ires-Cre* mice; scale bar, 200 μm; boxed insets depict regions analyzed in Figure 5.

(D) Aortic arch innervation density by indicated NJP neuron types was quantified (n = 5 or 6 mice; dorsal and/or ventral views independently quantified per mouse; mean ± SEM).

mice, enabling visualization of sensory terminals within internal organs such as the lung, stomach, and intestine. AAVs injected into NJP superganglia randomly infected 50%–60% of sensory neurons without labeling motor fibers of passage (Chang et al., 2015).

First, we sought to visualize the full repertoire of peripheral sensory endings in the aortic arch and carotid sinus (Figures 4A and S6). AAVs encoding a Cre-dependent tdTomato reporter (*AAV-flex-tdTomato*) were injected into the left NJP superganglia of *Vglut2-ires-Cre* mice, and histological analysis was performed. Whole-mount visualization of the aortic arch (Figure 4B) revealed the incoming aortic depressor nerve, which emanates as a fine branch from the superior laryngeal nerve (Figure S5A). The aortic depressor nerve contacts the peak of the aortic arch between the left common carotid and left subclavian arteries and bifurcates with each branch ramifying caudally across the dorsal or ventral surface in a saddle-like pattern. This innervation pattern matches previous descriptions of aortic arch innervation by bulk neuronal tracing approaches (Aumonier, 1972; Krauhs, 1979). Innervation of the right subclavian artery by sensory neurons from the right NJP ganglion was similarly observed (Figure S5B).

Furthermore, these approaches revealed a diversity of arterial terminal morphologies, including abundant flower-spray endings as well as rarer end-net terminals and glomus cell contacts (Figures 4C, 5A–5D, and S5C). Flower-spray endings are named based on their characteristic morphology consisting of punctate terminals densely packed into large (>30 μm) complex clusters. End-net endings are long, thin, and linear processes that emanate from the principal fiber tract. A third type of afferent was observed

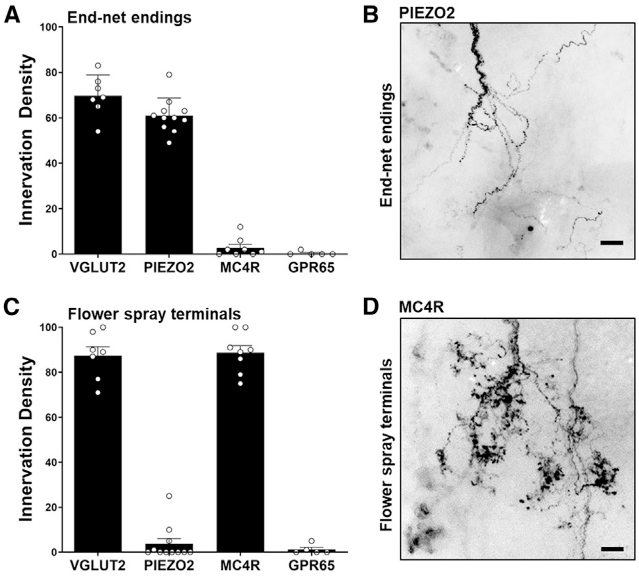

Figure 5. Genetic Access to Neurons with Different Aortic Terminal Types

NJP ganglia of *Vglut2-ires-Cre*, *Piezo2-ires-Cre*, *Mc4r-2a-Cre*, and *Gpr65-ires-Cre* mice were injected with both *AAV-flex-tdTomato* and *AAV-Gfp*, and aortic terminals were visualized using immunofluorescence.

(A and C) Innervation density of end-net endings (A) and flower-spray terminals (C) was calculated as a ratio of tdTomato signal/GFP signal times 100 (n = 5–11 mice; dorsal and/or ventral views independently quantified per mouse for C; mean ± SEM).

(B and D) Representative examples of end-net endings from PIEZO2 neurons (B) and flower-spray terminals from MC4R neurons (D) are depicted, derived from boxed insets of Figure 4C; scale bars, 30 μm.

See also Figures S5 and S6.

near aortic glomus cells, which serve as vascular chemosensors for respiratory gases. Small clusters of aortic glomus cells, like carotid glomus cells, express tyrosine hydroxylase, synaptophysin, and NDUFA4L2 (Dvorakova and Kummer, 2005; Zhou et al., 2016), and could be visualized by immunohistochemistry (Figures S5C–S5F). A similar diversity of neuronal terminals was observed in the carotid sinus (Figures S6A–S6F).

The physiological functions of flower-spray terminals and end-net endings have not been directly examined. We next sought genetic tools that enable selective manipulation of neurons that form each terminal type. We injected *AAV-flex-tdTomato* into NJP superganglia of various Cre knockin mice and examined aortic arch innervation using whole-mount fluorescence microscopy. We observed that both PIEZO2 and MC4R neurons densely innervated the aortic arch (Figures 4C and 4D); we note that these mouse lines also labeled sensory neurons that innervate other organs; for example, some neurons labeled in *Piezo2-ires-Cre* mice function as airway mechanoreceptors (Nonomura et al., 2017). Other vagal sensory neuron types labeled in *Gpr65-ires-Cre* and *Glp1r-ires-Cre* mice did not densely innervate the aorta.

For quantification of aortic terminal types, we simultaneously injected *AAV-flex-tdTomato* and a second AAV expressing a constitutive, Cre-independent GFP allele (*AAV-Gfp*) for normalization (Figures S6G and S6H). We counted the number of fluorescent flower-spray terminals and measured the fluorescence intensity of end-net endings and glomus cell contacts; we then expressed innervation density (ID) as the ratio of tdTomato/GFP measurements for each terminal type times 100 (Figure 5). In *Vglut2-ires-Cre* mice, we calculated IDs of 68.9 for end-net endings, 87.3 for flower-spray terminals, and 55.0 for glomus cell contacts, providing an upper technical limit for comparison. In *Piezo2-ires-Cre* mice, end-net endings were similarly labeled (ID = 60.9), but flower-spray terminals (ID = 2.8) and glomus cell contacts (ID = 4.4) were not. In contrast, in *Mc4r-2a-Cre* mice, flower-spray terminals were abundantly labeled (ID = 87.7), but end-net endings (ID = 2.6) and glomus cell contacts (ID = 6.2) were not. GPR65 neurons did not form any of these terminal types. We note that some PIEZO2 and MC4R neurons had fiber branches that passed through aortic and carotid bodies without local ramification (Figures S5G and S5H). Thus, within the context of the aortic depressor nerve, PIEZO2 neurons and MC4R neurons represent different aorta-innervating neuronal populations with distinct terminal morphologies. Moreover, mechanosensory terminals underlying the baroreceptor reflex, which are marked in *Piezo2-ires-Cre* mice, form end-net endings, but not flower-spray terminals or glomus cell contacts.

Loss of Aortic End-Net Terminals in *Piezo2-ABLATE* Mice

AAV-based anatomical mapping labels cells that express Cre in the adult, while DT-guided ablation potentially eliminates additional cells that express Cre transiently during development. Therefore, we directly examined which aortic sensory terminal types might be lost in *Piezo2-ABLATE* mice lacking a baroreceptor reflex. NJP ganglia of wild-type (non-ABLATE), *Piezo2-ires-Cre; loxP-DTR* and *Mc4r-2a-Cre; loxP-DTR* mice were simultaneously injected with DT and an AAV containing a constitutive mCherry allele for anatomical characterization. Flower-spray and end-net terminals were subsequently visualized in the aorta and quantified (Figure 6). *Piezo2-ABLATE* mice displayed a loss of aortic end-net endings but not flower-spray terminals, while *Mc4r-ABLATE* mice displayed an orthogonal decrease of flower-spray terminals but not end-net terminals. Thus, end-net terminals are selectively labeled in *Piezo2-ires-Cre* mice by AAV mapping and also specifically eliminated in *Piezo2-ires-Cre; loxP-DTR* mice following targeted neuron ablation.

Piezo2 Neurons Form Decorated Aortic Claws

Prior bulk tracing studies revealed that the aortic depressor nerve accesses the peak of the aortic arch, where dorsal and ventral branches separate and form dense terminals in a saddle-like pattern confined to the rostral aorta (Aumonier, 1972; Krauhs, 1979). This saddle-like fiber arrangement was similarly observed by AAV tracing approaches in *Vglut2-ires-Cre* mice and in *Mc4r-2a-Cre* mice, where the predominant flower-spray terminals are visualized.

PIEZO2 neurons, however, formed a distinct macroscopic architecture, which we describe as aortic claws that circumnavigate and grasp most of the aortic arch (Figures 4C and 7). This fiber organization was likely obscured in prior tracing studies because PIEZO2 neurons are a minority of sensory neurons in the aortic depressor nerve (Figure 7B). Incoming PIEZO2 neurites travel

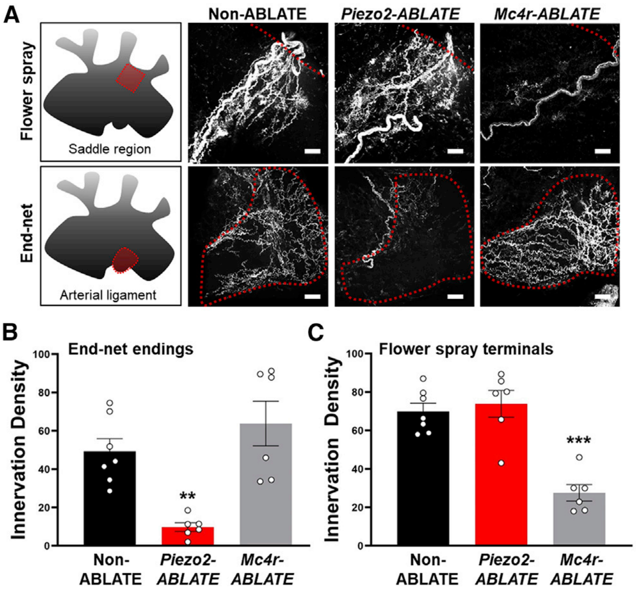

Figure 6. Loss of End-Net Terminals in *Piezo2-ABLATE* Mice

(A) NJP ganglia were simultaneously injected with DT for cell ablation and a Cre-independent AAV encoding mCherry for neuron visualization.

(A–C) Representative images and quantified innervation density (A) of flower-spray terminals (B) and end-net endings (C) from regions indicated; scale bars, 30 μm; (n = 6 or 7 mice; mean ± SEM; **$p < 0.005$ and ***$p < 0.0005$, ANOVA [Dunnett's multiple-comparison test]).

along the outer edge of the smooth muscle layer (Figure 7C), extend caudally beyond the saddle region, and form longer tracts with densest terminal deposition in the arterial ligament at the aortic base (Figures 7D and S7). PIEZO2 neuron claws are decorated with end-net terminals that emanate laterally and are dispersed along the rostral-caudal axis of the aortic arch. End-net terminals in the arterial ligament are beaded and labeled by immunohistochemistry for GFP (Figure S7) in *Piezo2-ires-Cre* mice; a *Piezo2-GFP* allele is knocked into the endogenous *Piezo2* locus along with the *ires-Cre* cassette resulting in a functional PIEZO2-GFP fusion protein (Woo et al., 2014). Genetically guided electron microscopy was performed to visualize PIEZO2 fibers labeled with a peroxidase targeted to mitochondria (Zhang et al., 2019). Cre-dependent peroxidase-encoding AAVs were injected into NJP ganglia of *Piezo2-ires-Cre* mice; the aorta was removed, stained with a peroxidase substrate, and analyzed by electron microscopy. PIEZO2 fibers in the arterial ligament were locally unmyelinated, bundled (five to ten neurons per bundle) and encompassed by individual Schwann cells, which in turn were embedded in collagen matrix (Figures 7E and 7F).

Dorsal and ventral branches of PIEZO2 neurons converge at the arterial ligament, and in some cases, the proximity of dorsal and ventral branch endings generated the appearance of an aortic ring, which can occasionally be observed by whole-mount analysis or in thick coronal aortic sections (Figure 7A). Aortic claws were not observed following AAV tracing of MC4R neurons. Sensory innervation of the arterial ligament is intact in *Phox2b-Cre; loxP-Piezo1; loxP-Piezo2* mice (Figures 7G and 7H), suggesting that sensory deficits in PIEZO knockout mice are not due to errant morphology of sensory terminals. By displaying a circumferential distribution of end-net terminals, PIEZO2 neurons are perfectly positioned to detect increases in arterial diameter with each pressure pulse.

DISCUSSION

Sensory neurons in the great vessels of the vascular system provide essential feedback for control of heart rate, blood pressure, and respiration. Baroreceptors detect momentary fluctuations in blood pressure, for example during movement or postural changes, and ensure real-time stabilization of cardiovascular output to ensure appropriate blood flow to the brain and body. Here, we used genetic approaches to reveal the architecture and organization of baroreceptor terminals. We found that PIEZO2 neurons of NJP ganglia, which represent a minority population of arterial sensory neurons, are the principal baroreceptors of the nervous system. Optogenetic activation of PIEZO2 neurons decreased blood pressure and heart rate, while ablating PIEZO2 neurons eliminated the baroreceptor reflex and the ability of the aortic depressor nerve to control heart rate and blood pressure. PIEZO2 neurons form end-net endings that adorn aortic claws, a striking macroscopic structure well suited to detect distension of the arterial wall. On the basis of these and previous knockout studies (Zeng et al., 2018), all major baroreceptors (1) are marked in *Piezo2-ires-Cre* mice, (2) use PIEZO1 and/or PIEZO2, and (3) are derived from sensory neurons of the nodose and inferior petrosal ganglia, which are the only sensory neurons that are both ablated following DT injection into NJP superganglia of *Piezo2-ires-Cre; loxP-DTR* mice, and also lack PIEZOexpression in *Phox2b-Cre*; *loxP-Piezo1*; *loxP-Piezo2* mice.

In principle, baroreflex deficits of *Phox2b-Cre*; *loxP-Piezo1*; *loxP-Piezo2* mice could be due to disrupted sensory transduction, errant morphology of sensory terminals, and/or altered neural circuit function following PIEZO deletion. Here, experiments involving electrical stimulation of the aortic depressor nerve demonstrated that the baroreceptor-responsive neural arc, from brainstem sensory terminals to motor output, is intact in *Phox2b-Cre*; *loxP-Piezo1*; *loxP-Piezo2* mice. Furthermore, anatomical tracing revealed that sensory neuron innervation of the arterial ligament was similar in *Phox2b-Cre*; *loxP-Piezo1*; *loxP-Piezo2* mice. These findings indicate that the PIEZO ion channels are not required for the morphological development of baroreceptor neurons or the function of downstream neural circuits. Instead, the loss of blood pressure sensing by the aortic depressor nerve in *Phox2b-Cre; loxP-Piezo1; loxP-Piezo2* mice is consistent with dysfunction of the signaling machinery that senses blood pressure.

Ablation of PIEZO2 neurons eliminated the baroreceptor reflex, while knockout of the *Piezo2* gene alone had no effect when *Piezo1* is preserved (Zeng et al., 2018), suggesting that at least some PIEZO2 neurons can depend on PIEZO1 for their function. The most parsimonious interpretation of these findings is that PIEZO1 and PIEZO2 are co-expressed within at least some baroreceptor afferents, where either is sufficient for blood pressure sensation. The extent of interaction between PIEZO1 and PIEZO2 in baroreceptors is unclear; for example, it is possible that they form mixed trimers with a 1:2 stoichiometry

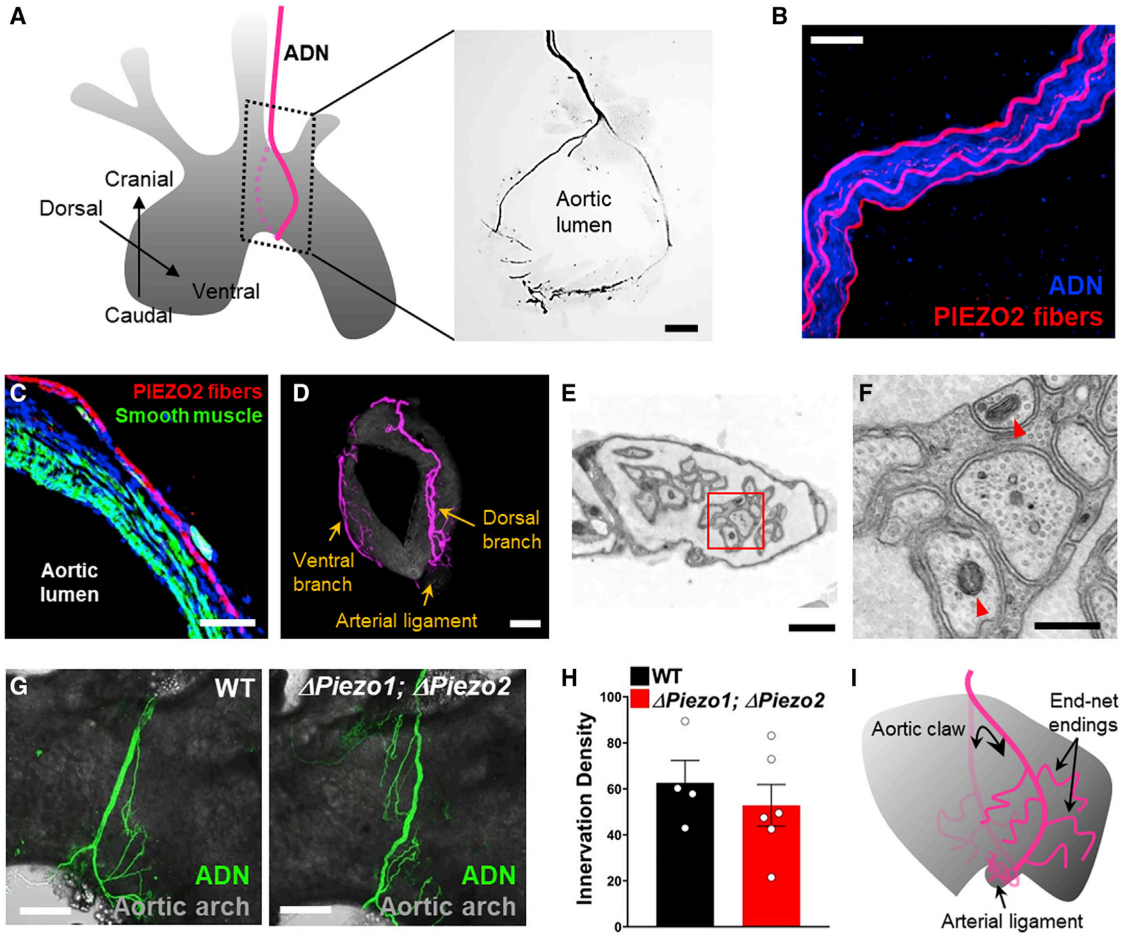

Figure 7. PIEZO2 Neurons Form Aortic Claws

(A) NJP ganglia of *Piezo2-ires-Cre* mice were injected with *AAV-flex-tdTomato*, and PIEZO2 fibers were visualized (right) by immunofluorescence in thick coronal aortic sections of regions depicted (left); scale bar, 100 μm. In this image, the proximity of dorsal and ventral branches of the aortic depressor nerve generates a ring-like morphology.

(B) PIEZO2 neurons visualized by confocal microscopy after AAV-guided mapping (red) represent a subset of fibers in the aortic depressor nerve, with all neurons visualized by immunofluorescence for synaptophysin (blue); scale bar, 20 μm.

(C) Immunochemistry to visualize PIEZO2 neurons (red), smooth muscle (Alexa 488-Phalloidin, green), and nuclei (TO-PRO-3, blue) in thin cryosections of aorta; scale bar, 25 μm.

(D) PIEZO2 neurites in a representative aorta after AAV mapping. Dense collaterals are observed in the arterial ligament at the aortic base; scale bar, 100 μm.

(E and F) Electron microscopy of PIEZO2 afferents in the arterial ligament. NJP ganglia from *Piezo2-ires-Cre* mice were injected with AAVs encoding a peroxidase targeted to the mitochondrial matrix; aortic arches were later harvested and stained, and the arterial ligament region was visualized by electron microscopy. Red arrowheads, peroxidase-labeled mitochondria; red box in (E) depicts region shown in (F); scale bars: 1 μm in (E) and 400 nm in (F).

(G and H) Representative images (G) and quantification (H) showing that neural innervation of the aortic arch (anti-neurofilament immunofluorescence, green) was similar in wild-type (WT) and *Phox2b-Cre; loxP-Piezo1; loxP-Piezo2* (Δ*Piezo1; ΔPiezo2*) mice; scale bar, 300 μm; n = 4–6 mice; mean ± sem.

(I) A cartoon depicting morphology of PIEZO2 neurons, which form aortic claws adorned with end-net terminals.

See also Figure S7.

or that they form independent complexes that operate in parallel. Furthermore, the relative expression levels of PIEZO1 and PIEZO2 may vary across baroreceptor terminals, leading to subtle differences in response properties, such as activation threshold or adaptation rate. At one extreme, it is possible that some neurons express PIEZO2 transiently during development or at low levels sufficient for Cre-mediated recombination, but rely primarily on PIEZO1 for force sensation. Despite such a potential for molecular heterogeneity, baroreceptor neurons marked in *Piezo2-ires-Cre* mice display a common architecture that suggests an absence of morphological heterogeneity.

Across physiological systems, mechanosensory neurons display a myriad of highly specialized terminal morphologies for force sensation. Hair cells of the auditory system detect sound vibration through displacement of stereocilia connected by tip links (Pan and Holt, 2015). Skin-innervating somatosensory neurons that underlie touch sensation display a diversity of terminal types, including corpuscular endings, Merkel cell

contacts, lanceolate endings, Ruffini endings, and free nerve endings (Abraira and Ginty, 2013). Proprioceptive endings include muscle spindle fibers that detect muscle length and Golgi tendon organs that detect muscle contractions (Proske and Gandevia, 2012). Other mechanosensory neurons detect organ stretch (Umans and Liberles, 2018), such as intraganglionic laminar endings in stomach muscle that detect gastric distension (Williams et al., 2016; Zagorodnyuk et al., 2001). Here, we report that baroreceptors detect force using a distinct terminal morphology: aortic claws with radially distributed and laterally projecting end-net endings. The anatomy of aortic baroreceptors perhaps shares some superficial resemblance to longitudinal lanceolate endings, which form circular endings that surround hair follicles and display perpendicularly emanating fibers (Abraira and Ginty, 2013). However, the aorta is much larger than a hair follicle, and baroreceptors detect pressure-induced increases in aortic diameter rather than orientation-selective hair follicle deflection. Thus, PIEZO2 neurons evolved a unique anatomical solution for the challenge of blood pressure sensation in the aortic wall.

Vagal neurons marked in *Mc4r-2a-Cre* mice form flower-spray terminals, the major arterial terminal type concentrated in the aortic arch saddle region. Future studies are needed into the functions of vagal MC4R neurons, as they appear to mediate a response distinct from classical baroreceptor and chemoreceptor reflexes of arterial sensory neurons. Additional classes of vagal afferents also innervate other sites in the cardiovascular system. Mechanoreceptors in the heart and veins detect increases in atrial filling and venous pressure to inform on total blood volume and, in response, induce a tachycardia response called the Bainbridge reflex (Hainsworth, 1991). Distinct cardiac sensory neurons function as chemoreceptors and/or mechanoreceptors and mediate a bradycardia response termed the Bezold-Jarisch reflex (Hainsworth, 1991). Optogenetic studies are particularly conducive for analysis of cardiovascular afferents, as selective stimulation of particular sensory neuron types using physiological approaches can otherwise be challenging because of the closed-loop nature of the cardiovascular system. Branch-selective optogenetic experiments revealed additional vagal sensory neurons that control heart rate and blood pressure, some of which are not confined to the aortic depressor nerve, not required for the baroreceptor reflex, and not marked in *Piezo2-ires-Cre* mice. We note that our studies were done under anesthesia with urethane, and it is possible that some physiological reflexes are sensitive to the anesthesia used. Finally, other blood vessel-innervating sensory neurons may not be detectable by our available Cre lines; we note that any additional aorta-innervating neuron types would not strongly affect blood pressure or heart rate on the basis of the blunted cardiovascular responses observed following aortic depressor nerve stimulation in *Piezo2-ABLATE* mice.

These aorta-to-brain sensory neuron types are distinct from vagal sensory neurons containing GLP1R and GPR65, which do not densely innervate the arterial wall. Vagal GLP1R neurons instead include gastrointestinal mechanosensors, while vagal GPR65 neurons innervate intestinal villi and stomach mucosa (Williams et al., 2016). Other vagal afferents control breathing, with some P2RY1 neurons receiving inputs from airway sensory cells clustered in neuroepithelial bodies (Chang et al., 2015). PIEZO2 expression marks several classes of sensory neurons, including baroreceptors, as well as airway stretch receptors (Kupari et al., 2019; Nonomura et al., 2017). Baroreception and airway mechanosensation involve different cohorts of PIEZO2 neurons, consistent with the differential consequence of branch-selective optogenetics in the aortic depressor nerve (which includes baroreceptors but not airway mechanosensors) and the vagal trunk distal to departure of the superior laryngeal nerve (which includes airway mechanosensors but not baroreceptors). Together, these findings extend our understanding of the functional diversity of sensory neuron types of the vagus nerve.

Altered structure and function of baroreceptor terminals occur during aging and hypertension, and decreased baroreflex sensitivity predicts risk for coronary artery disease and heart failure (La Rovere et al., 2008). Age- and diet-induced alterations in baroreflex sensitivity might be due to hardening of the elastic arterial wall, as well as to adaptations within peripheral and central neurons that participate in the baroreceptor reflex (Andresen et al., 1978; Angell-James, 1973; Wehrwein and Joyner, 2013). Extensive efforts to examine the structure of aortic neuron terminals after cardiovascular disease and hypertension have focused on alterations in saddle-like neurons dominated by flower-spray terminals, which we find here do not mediate baroreception. Selective analysis of aortic PIEZO2 neurons, and the end-net endings they form, during aging and atherosclerosis may provide new insights into dispositions for cardiovascular disease.

STAR★METHODS

Detailed methods are provided in the online version of this paper and include the following:

- KEY RESOURCES TABLE
- LEAD CONTACT AND MATERIALS AVAILABILITY
- EXPERIMENTAL MODEL AND SUBJECT DETAILS
- METHOD DETAILS
  - Ganglion injections of AAVs and DT
  - Immunohistochemistry
  - Optogenetic and electrical nerve stimulations
  - Physiological measurements
  - *In situ* hybridization
  - Electron microscopy
- QUANTIFICATION AND STATISTICAL ANALYSIS
- DATA AND CODE AVAILABILITY

SUPPLEMENTAL INFORMATION

Supplemental Information can be found online at https://doi.org/10.1016/j.celrep.2019.10.040.

ACKNOWLEDGMENTS

We thank Ardem Patapoutian and John Flanagan for manuscript comments, Bradford Lowell and Ardem Patapoutian for mice, Qiyu Zhang and David Ginty for help with electron microscopy, and Chen Ran for help with MATLAB scripts. The work was supported by NIH grants to S.D.L. (DP1 AT009497, R01 HL132255, and OT2 OD023848), a Faculty Scholars Award from the Howard Hughes Medical Institute to S.D.L. S.L.P. is an Open Philanthropy Fellow

of the Life Sciences Research Foundation. S.D.L. is an investigator of the Howard Hughes Medical Institute.

AUTHOR CONTRIBUTIONS

S.D.L., S.M., S.L.P., and R.B.C. designed and conceived the study. S.M., S.L.P., B.B., N.R.J., and D.E.S. performed anatomical tracing experiments. R.B.C. and S.M. performed optogenetics experiments. S.M. performed cell ablation studies, baroreflex measurements, and aortic depressor nerve stimulations. S.D.L. and S.M. wrote the manuscript.

DECLARATION OF INTERESTS

The authors declare no competing interests.

Received: May 23, 2019
Revised: September 13, 2019
Accepted: October 10, 2019
Published: November 19, 2019

REFERENCES

Abraira, V.E., and Ginty, D.D. (2013). The sensory neurons of touch. Neuron *79*, 618–639.

Andresen, M.C., and Kunze, D.L. (1994). Nucleus tractus solitarius—gateway to neural circulatory control. Annu. Rev. Physiol. *56*, 93–116.

Andresen, M.C., Krauhs, J.M., and Brown, A.M. (1978). Relationship of aortic wall and baroreceptor properties during development in normotensive and spontaneously hypertensive rats. Circ. Res. *43*, 728–738.

Angell-James, J.E. (1973). Characteristics of single aortic and right subclavian baroreceptor fiber activity in rabbits with chronic renal hypertension. Circ. Res. *32*, 149–161.

Aumonier, F.J. (1972). Histological observations on the distribution of baroreceptors in the carotid and aortic regions of the rabbit, cat and dog. Acta Anat. (Basel) *82*, 1–16.

Benarroch, E.E. (2008). The arterial baroreflex: functional organization and involvement in neurologic disease. Neurology *71*, 1733–1738.

Brown, A.M. (1980). Receptors under pressure. An update on baroreceptors. Circ. Res. *46*, 1–10.

Buch, T., Heppner, F.L., Tertilt, C., Heinen, T.J., Kremer, M., Wunderlich, F.T., Jung, S., and Waisman, A. (2005). A Cre-inducible diphtheria toxin receptor mediates cell lineage ablation after toxin administration. Nat. Methods *2*, 419–426.

Chang, R.B., Strochlic, D.E., Williams, E.K., Umans, B.D., and Liberles, S.D. (2015). Vagal sensory neuron subtypes that differentially control breathing. Cell *161*, 622–633.

Cheng, Z., Powley, T.L., Schwaber, J.S., and Doyle, F.J., 3rd. (1997). A laser confocal microscopic study of vagal afferent innervation of rat aortic arch: chemoreceptors as well as baroreceptors. J. Auton. Nerv. Syst. *67*, 1–14.

Dvorakova, M.C., and Kummer, W. (2005). Immunohistochemical evidence for species-specific coexistence of catecholamines, serotonin, acetylcholine and nitric oxide in glomus cells of rat and guinea pig aortic bodies. Ann. Anat. *187*, 323–331.

Fidone, S.J., and Sato, A. (1969). A study of chemoreceptor and baroreceptor A and C-fibres in the cat carotid nerve. J. Physiol. *205*, 527–548.

Hainsworth, R. (1991). Reflexes from the heart. Physiol. Rev. *71*, 617–658.

Kirchheim, H.R. (1976). Systemic arterial baroreceptor reflexes. Physiol. Rev. *56*, 100–177.

Krauhs, J.M. (1979). Structure of rat aortic baroreceptors and their relationship to connective tissue. J. Neurocytol. *8*, 401–414.

Kumada, M., Terui, N., and Kuwaki, T. (1990). Arterial baroreceptor reflex: its central and peripheral neural mechanisms. Prog. Neurobiol. *35*, 331–361.

Kupari, J., Haring, M., Agirre, E., Castelo-Branco, G., and Ernfors, P. (2019). An atlas of vagal sensory neurons and their molecular specialization. Cell Rep. *27*, 2508–2523.e4.

La Rovere, M.T., Pinna, G.D., and Raczak, G. (2008). Baroreflex sensitivity: measurement and clinical implications. Ann. Noninvasive Electrocardiol. *13*, 191–207.

Nonomura, K., Woo, S.H., Chang, R.B., Gillich, A., Qiu, Z., Francisco, A.G., Ranade, S.S., Liberles, S.D., and Patapoutian, A. (2017). Piezo2 senses airway stretch and mediates lung inflation-induced apnoea. Nature *541*, 176–181.

Pan, B., and Holt, J.R. (2015). The molecules that mediate sensory transduction in the mammalian inner ear. Curr. Opin. Neurobiol. *34*, 165–171.

Proske, U., and Gandevia, S.C. (2012). The proprioceptive senses: their roles in signaling body shape, body position and movement, and muscle force. Physiol. Rev. *92*, 1651–1697.

Ranade, S.S., Woo, S.H., Dubin, A.E., Moshourab, R.A., Wetzel, C., Petrus, M., Mathur, J., Bégay, V., Coste, B., Mainquist, J., et al. (2014). Piezo2 is the major transducer of mechanical forces for touch sensation in mice. Nature *516*, 121–125.

Rees, P.M. (1968). Electron microscopical observations on the architecture of the carotid arterial walls, with special reference to the sinus portion. J. Anat. *103*, 35–47.

Saito, M., Iwawaki, T., Taya, C., Yonekawa, H., Noda, M., Inui, Y., Mekada, E., Kimata, Y., Tsuru, A., and Kohno, K. (2001). Diphtheria toxin receptor-mediated conditional and targeted cell ablation in transgenic mice. Nat. Biotechnol. *19*, 746–750.

Schneider, C.A., Rasband, W.S., and Eliceiri, K.W. (2012). NIH Image to ImageJ: 25 years of Image Analysis. Nat. Methods *9*, 671–675.

Spyer, K.M. (1989). Neural mechanisms involved in cardiovascular control during affective behaviour. Trends Neurosci. *12*, 506–513.

Tränkner, D., Hahne, N., Sugino, K., Hoon, M.A., and Zuker, C. (2014). Population of sensory neurons essential for asthmatic hyperreactivity of inflamed airways. Proc. Natl. Acad. Sci. U S A *111*, 11515–11520.

Umans, B.D., and Liberles, S.D. (2018). Neural sensing of organ volume. Trends Neurosci. *41*, 911–924.

Wehrwein, E.A., and Joyner, M.J. (2013). Regulation of blood pressure by the arterial baroreflex and autonomic nervous system. Handb. Clin. Neurol. *117*, 89–102.

Williams, E.K., Chang, R.B., Strochlic, D.E., Umans, B.D., Lowell, B.B., and Liberles, S.D. (2016). Sensory neurons that detect stretch and nutrients in the digestive system. Cell *166*, 209–221.

Woo, S.H., Ranade, S., Weyer, A.D., Dubin, A.E., Baba, Y., Qiu, Z., Petrus, M., Miyamoto, T., Reddy, K., Lumpkin, E.A., et al. (2014). Piezo2 is required for Merkel-cell mechanotransduction. Nature *509*, 622–626.

Woo, S.H., Lukacs, V., de Nooij, J.C., Zaytseva, D., Criddle, C.R., Francisco, A., Jessell, T.M., Wilkinson, K.A., and Patapoutian, A. (2015). Piezo2 is the principal mechanotransduction channel for proprioception. Nat. Neurosci. *18*, 1756–1762.

Zagorodnyuk, V.P., Chen, B.N., and Brookes, S.J. (2001). Intraganglionic laminar endings are mechano-transduction sites of vagal tension receptors in the guinea-pig stomach. J. Physiol. *534*, 255–268.

Zeng, W.Z., Marshall, K.L., Min, S., Daou, I., Chapleau, M.W., Abboud, F.M., Liberles, S.D., and Patapoutian, A. (2018). PIEZOs mediate neuronal sensing of blood pressure and the baroreceptor reflex. Science *362*, 464–467.

Zhang, Q., Lee, W.A., Paul, D.L., and Ginty, D.D. (2019). Multiplexed peroxidase-based electron microscopy labeling enables simultaneous visualization of multiple cell types. Nat. Neurosci. *22*, 828–839.

Zhou, T., Chien, M.S., Kaleem, S., and Matsunami, H. (2016). Single cell transcriptome analysis of mouse carotid body glomus cells. J. Physiol. *594*, 4225–4251.

STAR★METHODS

KEY RESOURCES TABLE

REAGENT or RESOURCE	SOURCE	IDENTIFIER
Antibodies		
anti-Synaptophysin (Guinea pig)	Synaptic systems	Cat#101-004; RRID: AB_1210382
anti-DTR (Goat, also known as HB-EGF)	Fisher scientific	Cat#AF259NA; RRID: AB_354429
anti-GFP (Chicken)	Aves Labs	Cat#GFP-1020; RRID: AB_10000240
anti-tdTomato (Rabbit)	Rockland Immunochemicals	Cat#600401379; RRID: AB_2209751
anti-Tyrosine hydroxylase (Rabbit)	Millipore Sigma	Cat#AB152; RRID: AB_390204
anti-Neurofilament (Rabbit)	BioLegend	Cat#841001; RRID: AB_2565457
Bacterial and Virus Strains		
AAV9-DIO-matrix-dAPEX2	Zhang et al., 2019	N/A
AAV-flex-tdTomato	Addgene	Cat#51502-AAV9
AAV-Gfp	Addgene	Cat#105542-AAV9
AAV-mCherry	Addgene	Cat#105544-AAV9
Chemicals, Peptides, and Recombinant Proteins		
Diphtheria toxin	Sigma	Cat#D0564
Fast Green FCF Dye	Sigma	Cat#F7252
Phenylephrine	Sigma	Cat#P6126-25G
Critical Commercial Assays		
RNAscope Fluorescent Multiplex Assay kit	ACDBio	Cat#320850
Deposited Data		
Raw images for quantification of neurons and terminals	Mendeley data	https://doi.org/10.17632/3csd97w5wj.1
All the raw data points for graphs	Mendeley data	https://doi.org/10.17632/3csd97w5wj.1
Experimental Models: Organisms/Strains		
Gpr65-ires-Cre	Chang et al., 2015; Williams et al., 2016	Deposited in Jackson laboratory (Cat#029282)
Glp1r-ires-Cre	Chang et al., 2015; Williams et al., 2016	Deposited in Jackson laboratory (Cat# 029283)
Phox2b-Cre; loxP-Piezo1; loxP-Piezo2	Zeng et al., 2018	N/A
Vglut2-ires-Cre	Bradford Lowell (Beth Israel Deaconess Medical Center)	N/A
wild type C57BL/6J	Jackson laboratory	Cat#000664
Piezo2-EGfp-ires-Cre	Jackson laboratory	Cat#027719
Mc4r-2a-Cre	Jackson laboratory	Cat#030759
LoxP-ChR2	Jackson laboratory	Cat#012569
LoxP-DTR	Jackson laboratory	Cat#007900
Oligonucleotides		
Piezo2 probe (Channel1)	ACDBio	Cat#500501
Vglut2 probe (Channel 2)	ACDBio	Cat#319171-C2
Software and Algorithms		
ImageJ (FIJI program)	Schneider et al., 2012	https://imagej.nih.gov/ij/
MATLAB (R2018a)	MathWorks	N/A
Prism 8	Graphpad	N/A
Acqknowledge 5 software	BIOPAC systems, Inc.	Cat#ACK100W

LEAD CONTACT AND MATERIALS AVAILABILITY

Further information and requests for resources and reagents should be directed and will be fulfilled by the Lead Contact, Stephen Liberles (Stephen_Liberles@hms.harvard.edu). Mouse lines used in this study were derived from crosses involving previously published mouse lines with availability information below, and this study did not generate other unique reagents.

EXPERIMENTAL MODEL AND SUBJECT DETAILS

All animal husbandry and procedures were performed in compliance with institutional animal care and committee guidelines. *Gpr65-ires-Cre*, *Glp1r-ires-Cre*, and *Phox2b-Cre; loxP-Piezo1; loxP-Piezo2* mice were described before (Chang et al., 2015; Williams et al., 2016; Zeng et al., 2018); *Vglut2-ires-Cre* mice were a generous gift from Bradford Lowell (Beth Israel Deaconess Medical Center); and wild-type C57BL/6J (000664), *Piezo2-EGfp-ires-Cre* (027719), *Mc4r-2a-Cre* (030759), *LoxP-ChR2* (012569), and *LoxP-DTR* (007900) mice were purchased (Jackson). Male and female mice between 8-16 weeks old were used for all studies, and no differences based on sex were observed.

METHOD DETAILS

Ganglion injections of AAVs and DT

AAV-flex-tdTomato (Addgene, 51502-AAV9), *AAV-Gfp* (Addgene, 105542-AAV9), and *AAV-mCherry* (Addgene, 105544-AAV9) were purchased. Surgically exposed NJP superganglia were serially injected (10 X 13.8 nl) with AAV injection solution (AAV titer > 6.7 × 10^{12} vg/ml and 0.05% Fast Green FCF Dye, Sigma) or DT injection solution (5 μg/ml DT Sigma D0564, 0.05% Fast Green FCF Dye, PBS) using a Nanoject Injector (Drummond). Dye typically filled the NJP ganglion, but occasionally, the injection needle was repositioned for maximal dye spread. In control experiments, ectopic AAV infection of superior cervical ganglia was not observed by fluorescence microscopy. After AAV infection, animals were sacrificed four weeks later for histological analysis. After DT injection, animals were used at least two weeks later for physiological analysis, and the extent of ablation was analyzed post hoc by DTR immunostaining of NJP ganglia.

Immunohistochemistry

Tissue was obtained after fixation by intracardial perfusion (5 mL PBS then 5 mL 10% neutral buffered formalin or NBF in PBS, Sigma). NJP superganglia and cardiac tissue including the heart, aorta, and carotid sinus, were dissected, fixed overnight (10% NBF, PBS, 4°C), washed (3X PBS), and cleaned of periaortic fat, small vessels and other surrounding tissue. For whole mount analysis (except Figures 4B and S4A), tissue was permeabilized (11.5 g glycine, 400 mL PBS with 0.2% Triton-X, 100 mL DMSO, 37°C, 1 week), incubated with blocking buffer [5% donkey serum, Jackson 017-000-121, in PBS with 0.05% Tween-20 (PBST), RT, 1 h], and incubated with primary antibody (1:200 in blocking buffer, 4°C, overnight). Primary antibodies used were anti-Synaptophysin (Guinea pig, 101-004, Synaptic systems), anti-DTR (also known as HB-EGF, Goat, AF259NA, Fisher scientific), anti-GFP (Chicken, GFP-1020, Aves Labs), anti-tdTomato (Rabbit, 600401379, Rockland Immunochemicals), anti-Tyrosine hydroxylase (Rabbit, AB152, Millipore Sigma), and anti-Neurofilament (Rabbit, 841001, BioLegend). Samples were then washed (4x PBST, RT, 10 min), incubated with fluorescent secondary antibodies (Jackson Immunoresearch, 1:200-500, PBST, RT, 2 h), and washed again (4x PBST, RT, 10 min). Tissue was mounted between two thin coverglasses in Fluoromount-G medium (200 μl, SouthernBiotech) and visualized by fluorescence microscopy. Aortic claws were additionally visualized (Figures 6A and 6D) in thick coronal sections of aorta after staining as above. In Figure 6C, a similar protocol was followed for immunohistochemistry of cryosections, except after overnight fixation, tissue was cryosectioned and washed prior to blocking, and was not permeabilized. Images in Figure 4B and S4A involved native fluorescence in unfixed tissue.

Optogenetic and electrical nerve stimulations

Optogenetics experiments were performed as described (Chang et al., 2015), with minor modification of illumination parameters (5 ms pulses, 3-5 mW intensity, 20 Hz frequency, 10 s duration). For electrical stimulation (4-20 Hz frequency, 2 ms pulses, 10 V intensity, 20 s duration), the aortic depressor nerve was identified as a thin fiber emanating from the superior laryngeal nerve beneath a characteristic fat deposit, and placed on bipolar platinum electrodes connected to an electrical stimulator (Grass Instruments, SD5).

Physiological measurements

Mice were anesthetized with urethane, and body temperature maintained at 37°C with a heating pad. Heart rate was recorded using the ECG100C electrocardiogram amplifier (Biopac, ECG100C), and blood pressure was recorded in anesthetized mice as done previously (Zeng et al., 2018). The baroreceptor reflex was evoked by intravenous administration of phenylephrine (50 μl, 1 mg/ml, PBS) via a polyethylene catheter (Instech Laboratories) cannulated into the left femoral vein. Electrograms for blood pressure and heart rate were acquired and, for heart rate transformed to beats per minute over time, using the Acqknowledge program (ver. 5.0.1).

In situ hybridization

Single and two-color *in situ* hybridization (Figure S3) was performed on cryosections (10 μm) of freshly frozen NJP ganglia using the RNAscope Fluorescent Multiplex Assay kit (ACDBio). RNAscope Probes were used as *Piezo2* (500501, Channel1, ACDBio), *Vglut2* (319171-C2, Channel 2, ACDBio). For Figure S3A, immunohistochemistry for DTR was performed subsequent to *in situ* hybridization using the above protocol without fixation.

Electron microscopy

NJP ganglia of *Piezo2-ires-Cre* mice were injected with a Cre-dependent AAV (*AAV9-DIO-matrix-dAPEX2*) that targets a peroxidase (dAPEX2) to the mitochondria through a matrix localization sequence from cytochrome *c* oxidase subunit 4 (COX4) (Zhang et al., 2019). Three weeks later, aortas were harvested after intracardial perfusion of fixative (2% formaldehyde, 2.5% glutaraldehyde, cacodylate buffer), fixed in fixative (overnight, 4°C), washed (1x10 min cacodylate buffer, 2x10 min cacodylate buffer with 50 mM glycine, 2x10 min cacodylate buffer), and stained for peroxidase activity in a 96 well plate (0.3 mg/ml 3,3′-Diaminobenzidene tetrahydrochloride hydrate, 0.003% hydrogen peroxide, cacodylate buffer, 1 hr, RT). Cacodylate buffer is 0.15 M sodium cacodylate, 0.04% Calcium chloride, pH 7.4. The arterial ligament was removed, washed (4x10 min cacodylate buffer), fixed (3% glutaraldehyde, cacodylate buffer, overnight, 4°C), washed (1x10 min cacodylate buffer), incubated (1% osmium tetroxide, 1.5% potassium ferrocyanide, 1 hr, RT), washed (2x10 min water, 1x10 min in 50 mM Maleic acid, pH 5.15), incubated (1 hr, RT) in 1% uranyl acetate (in 50 mM Maleic acid, pH 5.15), washed (2x10 min water), and dehydrated in grades of alcohol (10 min each; 50%, 70%, 90%, 100%, 100% ethanol in water). Samples were incubated (1 hr, RT) in neat propyleneoxide, incubated (overnight, 4°C) with 1:1 propyleneoxide: TAAB Epon (TAAB Laboratories Equipment, Ltd.), and incubated with TAAB Epon (4°C, 48 hours) for resin polymerization. Ultrathin sections (~60 nm) were cut on a Reichert Ultracut-S microtome, picked up on copper grids, stained with 0.2% lead citrate, and examined on a TecnaiG^2 Spirit BioTWIN electron microscope. Images were recorded with an AMT 2k CCD camera.

QUANTIFICATION AND STATISTICAL ANALYSIS

Sample sizes are provided in each Figure Legend; each sample is derived from a different animal, except for data in Figures 4D and 5C where dorsal and/or ventral views from the same mouse were independently quantified. Data in graphs are represented as mean ± sem. All tests of statistical significance were performed using one-way ANOVA Dunnett's multiple comparison tests on Prism 8 software (Graphpad), and involved comparisons with WT mice (Figures 2 and 3), non-ABLATE mice (Figure 6), and control mice (Figure S4).

For quantification of innervation density, flower spray terminals were defined as localized clusters of endings with a cumulative diameter greater than 30 μm. Fluorescence intensity in end-net endings was quantified (FIJI program, based on ImageJ) in aortic regions devoid of flower spray terminals, with flower sprays visualized using a Cre-independent fluorophore. Innervation density of glomus cell contacts was calculated (FIJI program, based on ImageJ) using all fluorescent pixels overlapping synaptophysin-labeled glomus cells, including fibers of passage.

Heart rate and blood pressure were quantified as the integrated response during the stimulation period (optogenetics: 10 s, electrical stimulation: 20 s, phenylephrine injection: first 10 s after injection), with blood pressure represented as the average of the integrated changes in systolic and diastolic pressures. Percentage changes (%Δ) in heart rate and blood pressure were expressed relative to the immediate pre-stimulus measurement of the same duration. Physiological parameters were quantified using MATLAB (R2018a, MathWorks), and graphed and examined for statistical significance using Prism 8 software (Graphpad).

DATA AND CODE AVAILABILITY

Primary images and data obtained during this study are available at Mendeley, https://doi.org/10.17632/3csd97w5wj.1

Cell Reports, Volume *29*

Supplemental Information

Arterial Baroreceptors Sense Blood Pressure through Decorated Aortic Claws

Soohong Min, Rui B. Chang, Sara L. Prescott, Brennan Beeler, Narendra R. Joshi, David E. Strochlic, and Stephen D. Liberles

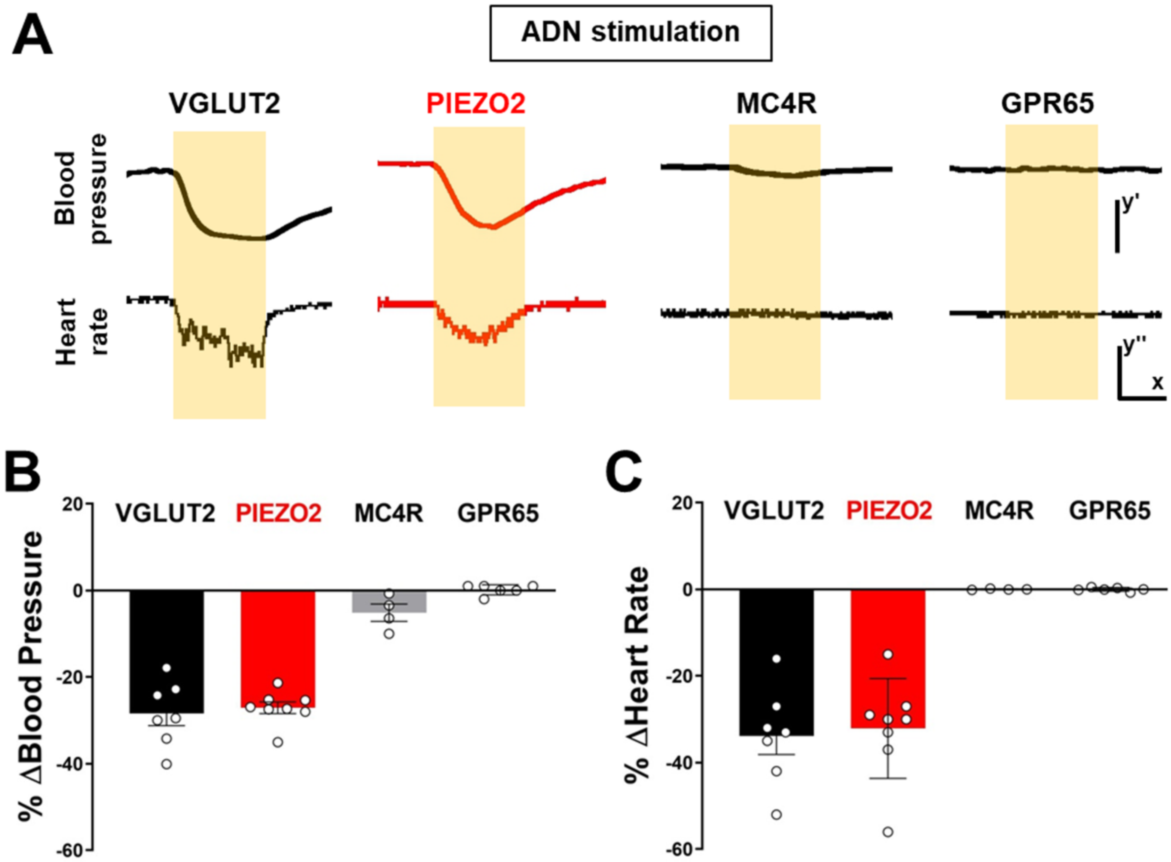

Figure S1. Optogenetic activation of sensory neurons in the aortic depressor nerve (Related to Figure 1). (A) Representative traces of blood pressure and heart rate with optogenetic stimulation (yellow shading) of the aortic depressor nerve in VGLUT2 (*Vglut2-ires-Cre; loxP-ChR2*), PIEZO2 (*Piezo2-ires-Cre; loxP-ChR2*, red), MC4R (*Mc4r-2a-Cre; loxP-ChR2*), and GPR65 (*Gpr65-ires-Cre; loxP-ChR2*) mice, scale bars: y': 20 mmHg, x: 5 sec, y'': 100 BPM. Quantifying changes in blood pressure (B) and heart rate (C) following illumination of the aortic depressor nerve in mice indicated, n: 4-8, mean ± sem.

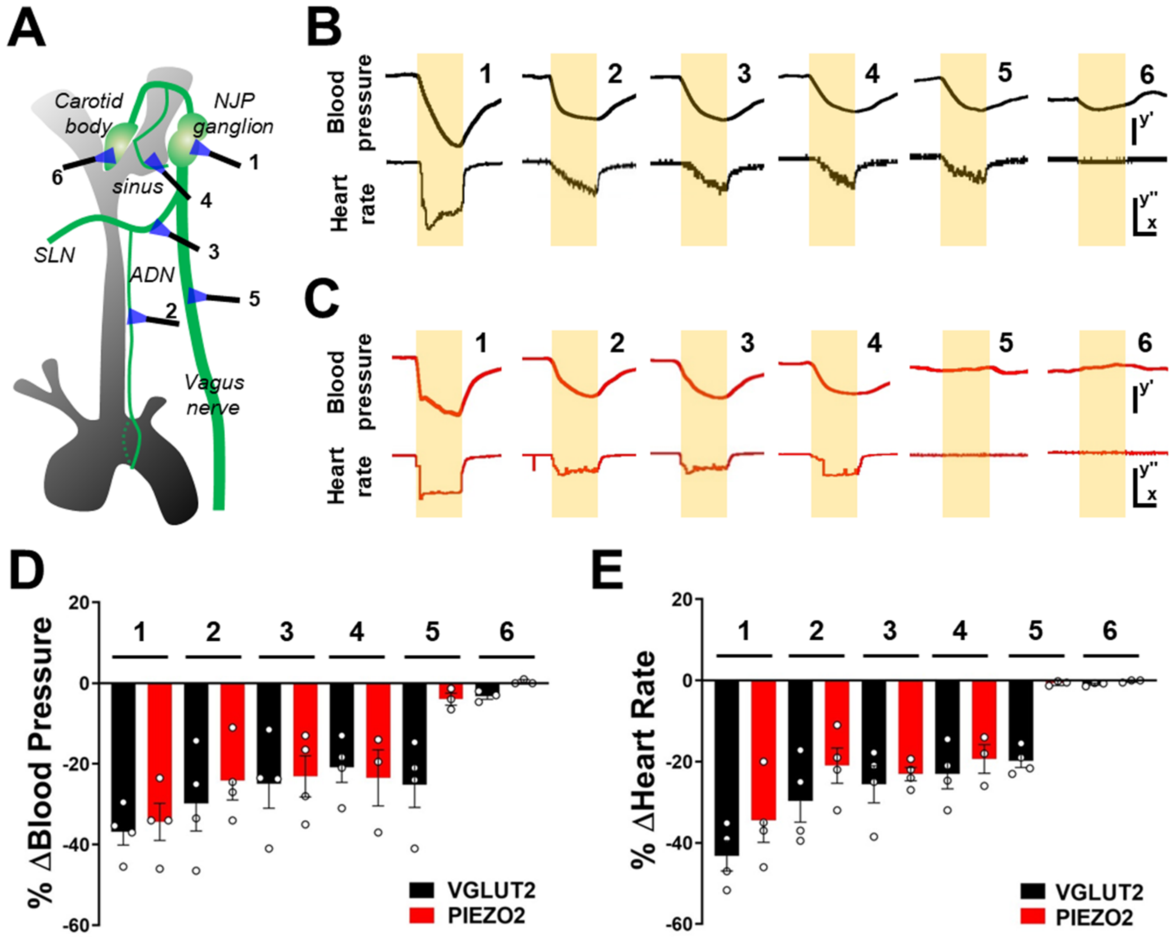

Figure S2. Nerve branch-selective optogenetics (Related to Figure 1). (A) Cartoon depicting various sites of optogenetic stimulation, including 1: NJP ganglion, 2: aortic depressor nerve (ADN), 3: superior laryngeal nerve (SLN), 4: carotid sinus, 5: vagal trunk after departure of SLN, and 6: carotid body. Representative traces of blood pressure and heart rate with optogenetic stimulation (yellow shading) occurring at sites indicated (1-6 from panel A) in *Vglut2-ires-Cre; loxP-ChR2* (B, black) and *Piezo2-ires-Cre; loxP-ChR2* (C, red) mice, scale bars: y': 20 mmHg, x: 5 sec, y'': 100 BPM. Quantifying changes in blood pressure (D) and heart rate (E) following optogenetic stimulation of *Vglut2-ires-Cre; loxP-ChR2* (black) and *Piezo2-ires-Cre; loxP-ChR2* (red) mice, n: 3-4, mean ± sem).

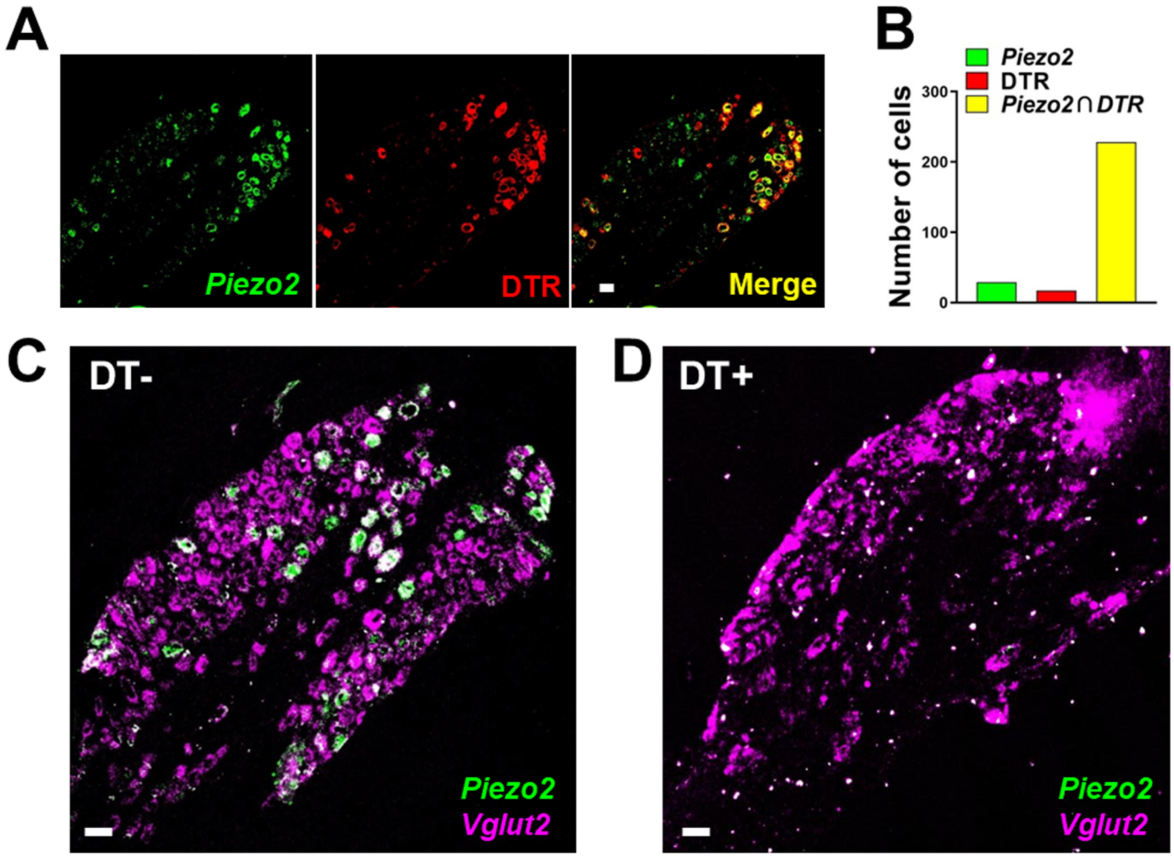

Figure S3. Assessing specificity of PIEZO2 neuron targeting in *Piezo2-ABLATE* mice (Related to Figure 2). (A) Sequential *in situ* hybridization for *Piezo2* transcript (green) and immunochemistry for DTR (red) in cryosections of vagal ganglia from *Piezo2-ires-Cre; loxP-DTR* mice, scale bar: 20 µm. (B) Counts of cells expressing only *Piezo2* (green), only DTR (red), or both (yellow), n: 5 sections from 2 mice. Two color *in situ* hybridization using cRNA probes that recognize *Piezo2* (green) and *Vglut2* (magenta) in cryosections of vagal ganglia from *Piezo2-ires-Cre; loxP-DTR* mice treated (C) without or (D) with DT, scale bar: 30 µm.

A

Blood pressure (mmHg)	Control	*Piezo2-ABLATE* (Bilateral)	*Piezo2-ABLATE* (Unilateral)
Mean	66.33	69.92	69.43
Standard Error	±4.775	±2.237	±4.657

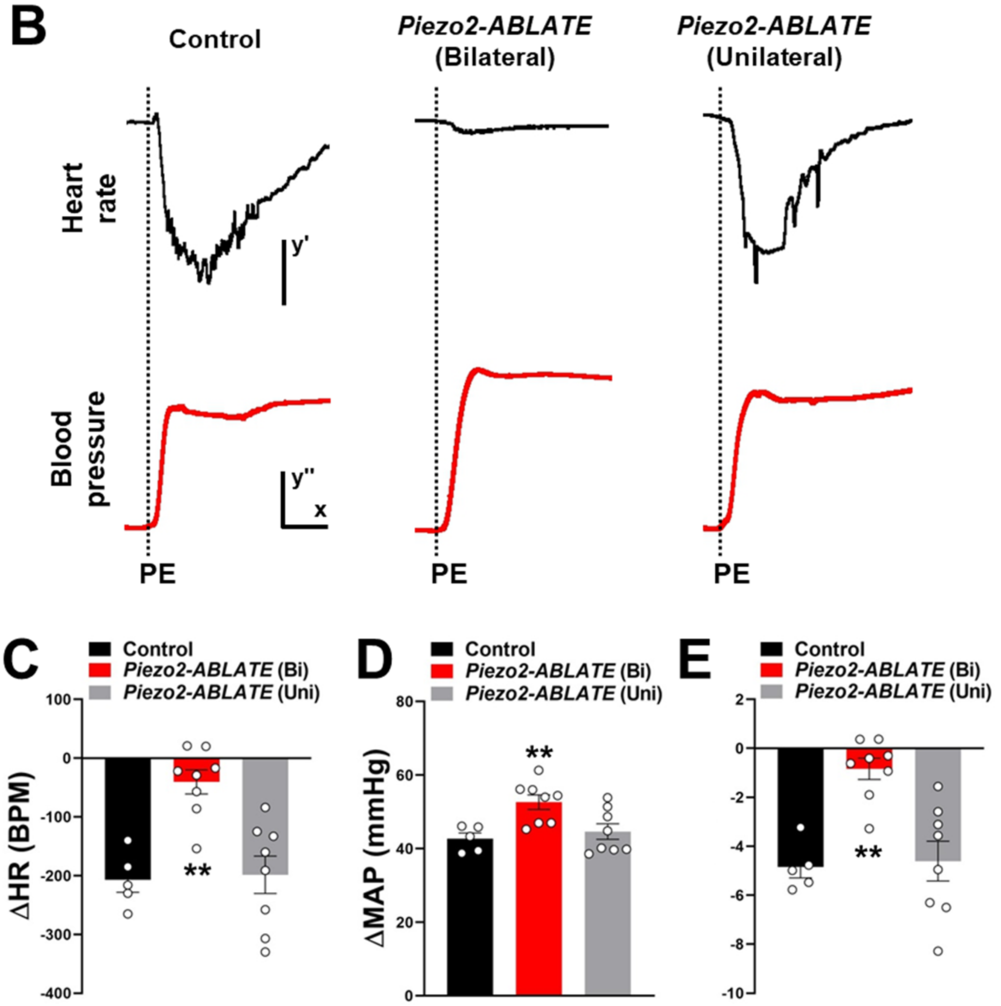

Figure S4. Loss of baroreceptor reflex requires bilateral ablation of PIEZO2 neurons in NJP ganglia (Related to Figure 2). (A) Resting blood pressure of *loxP-DTR* mice injected bilaterally with DT (control) and *Piezo2-ires-Cre; loxP-DTR* mice injected unilaterally or bilaterally with DT. (B) Assessment of baroreflex integrity in *loxP-DTR* mice injected bilaterally with DT (control), and *Piezo2-ires-Cre; loxP-DTR* mice injected unilaterally or bilaterally with DT. Representative effects of phenylephrine injection (dashed line) on blood pressure and heart rate, scale bars, y': 100 BPM, x: 10 sec, y'': 20 mmHg. Quantification of phenylephrine (PE)-induced change in heart rate or HR (C), change in mean arterial blood pressure or MAP (D), and baroreflex (E), defined as change in HR (Δ BPM) divided by change in BP (Δ mmHg), n: 5-8, mean ± sem, **p<.005.

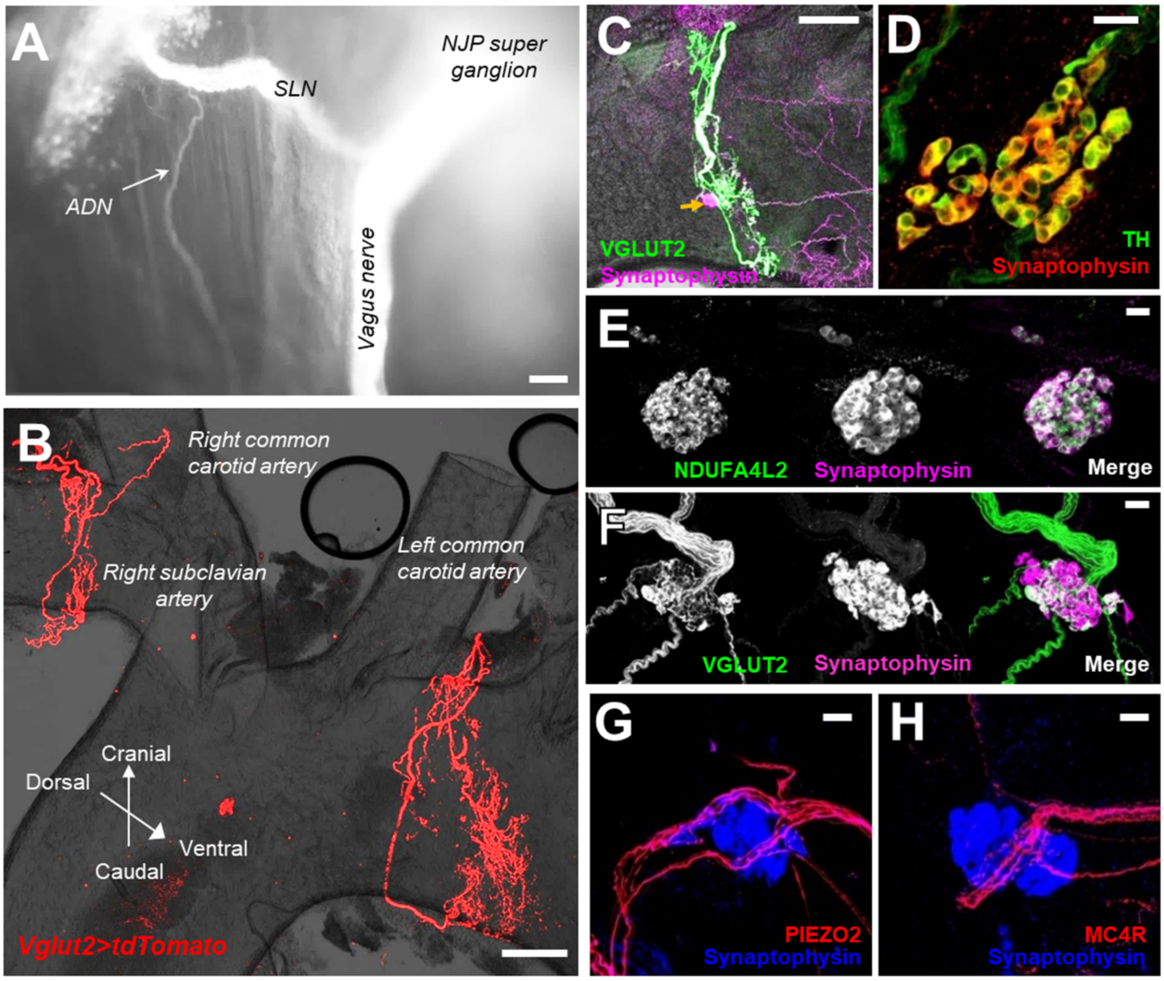

Figure S5. Visualizing innervation of the carotid sinus (Related to Figure 5). (A) Wholemount image of native fluorescence from the aortic depressor nerve (ADN) and superior laryngeal nerve (SLN) after ganglion injection of *AAV-Gfp* in wild type mice, scale bar 100 µm. (B) Wholemount anti-tdTomato immunofluorescence of the great aortic vessels in *Vglut2-ires-Cre; loxP-tdTomato* mice, scale bar 300 µm. (C) Aortic arch immunohistochemistry for tdTomato (green) and synaptophysin (magenta) after injection of *AAV-flex-tdTomato* into NJP ganglia of *Vglut2-ires-Cre* mice, yellow arrow indicates location of an aortic body, scale bar 300 µm. (D) Aortic arch immunohistochemistry for tyrosine hydroxylase (green) and synaptophysin (red), scale bar 20 µm. (E) Aortic body immunohistochemistry for NDUFA4L2 (green) and synaptophysin (magenta), scale bar 20 µm. (F) Aortic body immunohistochemistry for tdTomato (green) and synaptophysin (magenta) after injection of *AAV-flex-tdTomato* into NJP ganglia of *Vglut2-ires-Cre* mice, scale bar 20 µm. Two color immunohistochemistry for synaptophysin (blue) and tdTomato (red) to visualize neuronal fibers of passage labeled by injection of *AAV-flex-tdTomato* into NJP ganglia of *Piezo2-ires-Cre* mice (G) and *Mc4r-2a-Cre* mice (H), scale bar 20 µm.

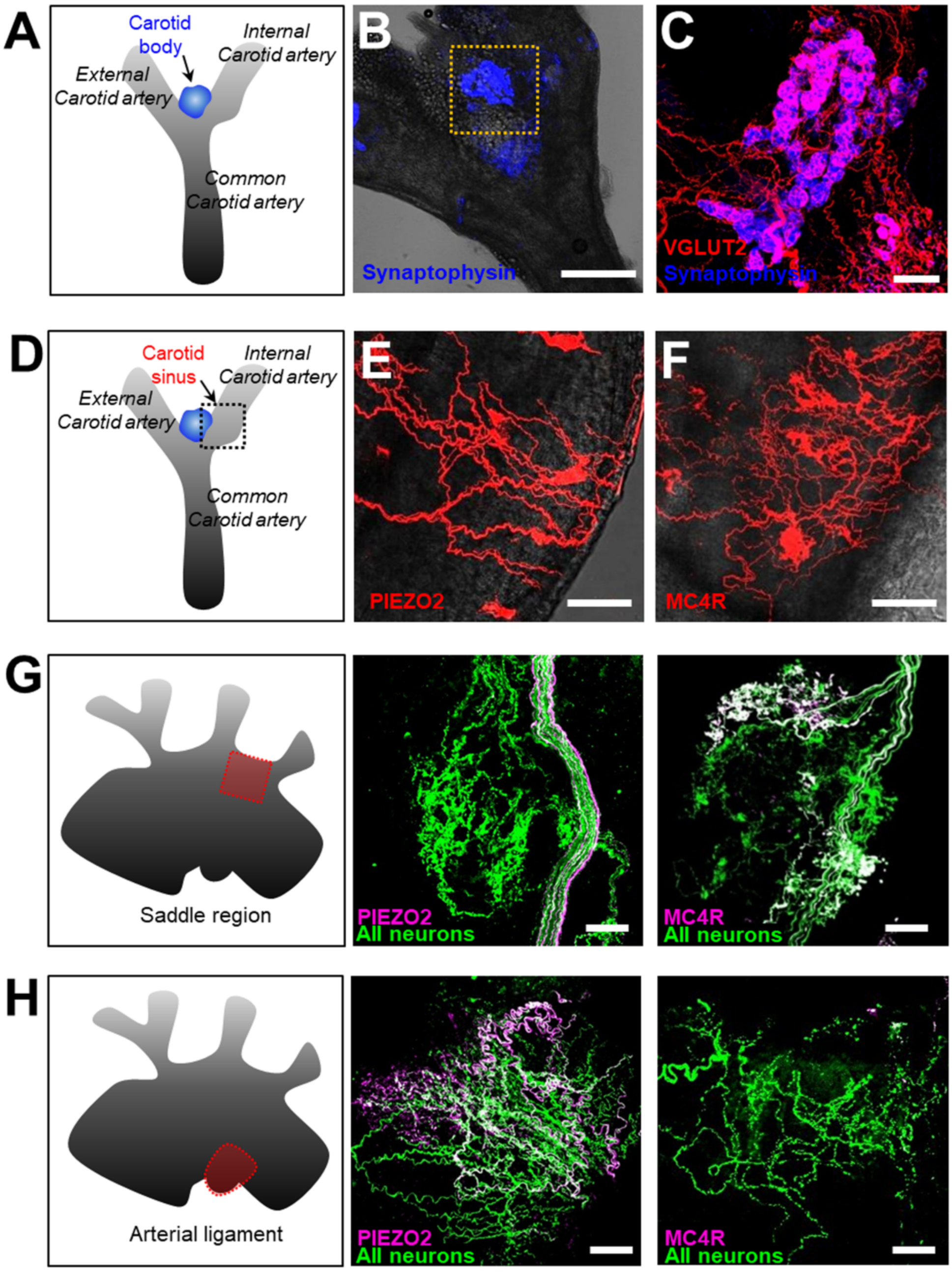

Figure S6. Neuron subtypes that innervate the carotid sinus and aortic arch (Related to Figure 5). (A, D) Cartoon depictions of the carotid sinus. Carotid glomus cells can be visualized (blue) by immunostaining for synaptophysin (B-C). Vagal afferents were visualized by immunochemistry for tdTomato (red) following injection of *AAV-flex-tdTomato* into NJP ganglia of *Vglut2-ires-Cre* mice (C), *Piezo2-ires-Cre* mice (E), and *Mc4r-2a-Cre* mice (F), scale bars: 400 µm for B; 20 µm for C; 100 µm for E-F. Boxed region in B depicts regions of analysis for panels C, while boxed region in D depicts region of analysis for E-F. Representative images used for quantitative analysis of flower spray terminals in the aortic saddle region (G) and end-net endings in the arterial ligament (H). Vagal afferents were visualized by immunochemistry following injection of Cre-independent *AAV-Gfp* (green, ALL NEURONS) and *AAV-flex-tdTomato* (magenta) into NJP ganglia of *Piezo2-ires-Cre* (PIEZO2) or *Mc4r-2a-Cre* (MC4R) mice, scale bar 30 µm.

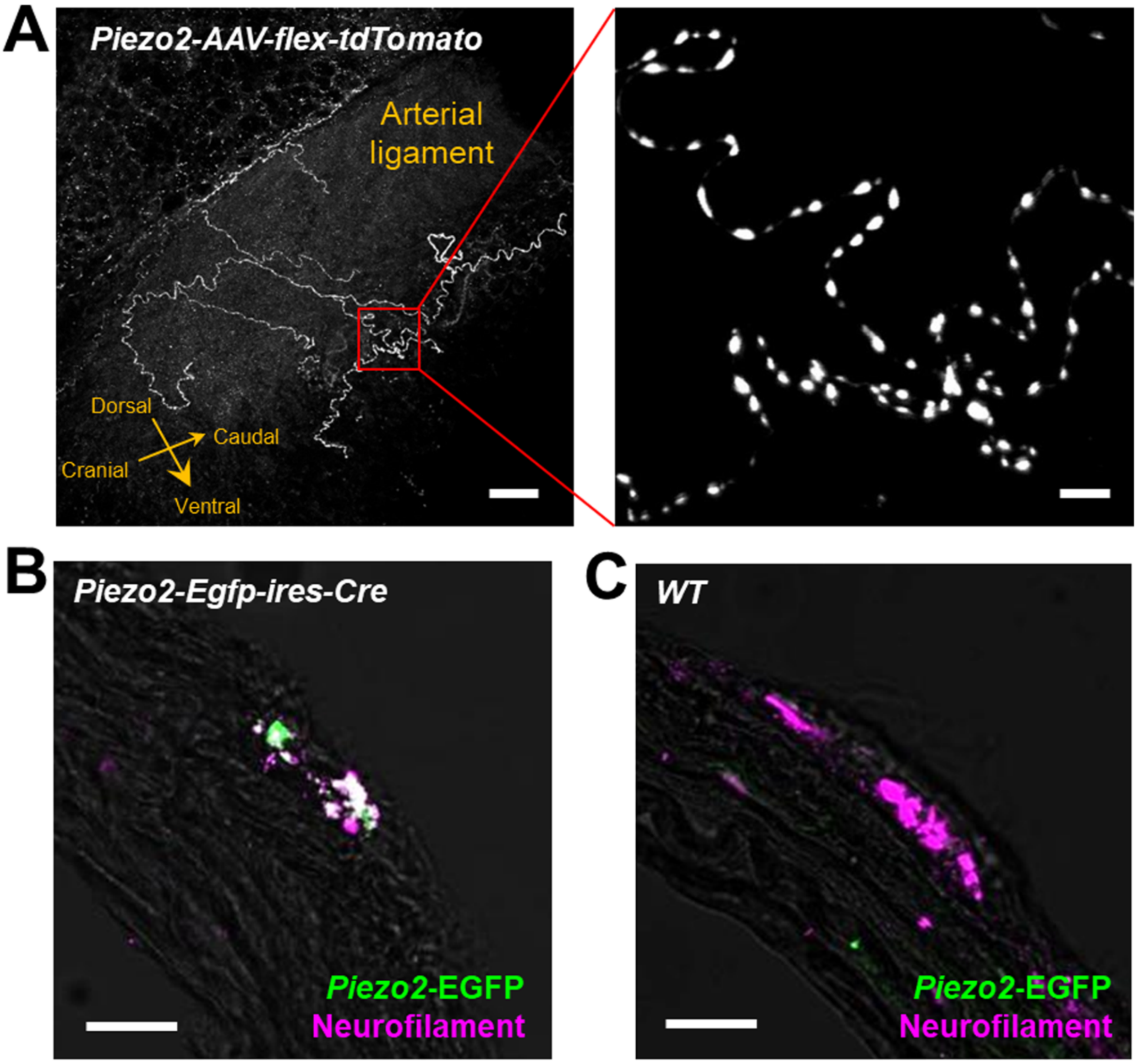

Figure S7. Sensory neuron innervation of the arterial ligament (Related to Figure 7). (A) Vagal afferents were visualized in the arterial ligament by immunochemistry for tdTomato following injection of *AAV-flex-tdTomato* into NJP ganglia of *Piezo2-ires-Cre* mice, scale bars, left: 30 µm, right: 10 µm. The right panel is a high magnification, partial Z-stack image from a region (red box) of the left image. Immunohistochemistry for GFP (green) and neurofilament (magenta) in arterial ligament of (B) *Piezo2-ires Cre* and (C) wild type mice. *Piezo2-ires-Cre* mice express a PIEZO2-GFP fusion protein from the endogenous *Piezo2* locus, scale bars: 10 µm.
